# Efficient in vivo base editing via single adeno-associated viruses with size-optimized genomes encoding compact adenine base editors

**DOI:** 10.1038/s41551-022-00911-4

**Published:** 2022-07-28

**Authors:** Jessie R. Davis, Xiao Wang, Isaac P. Witte, Tony P. Huang, Jonathan M. Levy, Aditya Raguram, Samagya Banskota, Nabil G. Seidah, Kiran Musunuru, David R. Liu

**Affiliations:** 1 Merkin Institute of Transformative Technologies in Healthcare, Broad Institute of MIT and Harvard, Cambridge, MA USA; 2 Department of Chemistry and Chemical Biology, Harvard University, Cambridge, MA USA; 3 Howard Hughes Medical Institute, Harvard University, Cambridge, MA USA; 4 Cardiovascular Institute, Perelman School of Medicine at the University of Pennsylvania, Philadelphia, PA USA; 5 Division of Cardiovascular Medicine, Department of Medicine, Perelman School of Medicine at the University of Pennsylvania, Philadelphia, PA USA; 6 Department of Genetics, Perelman School of Medicine at the University of Pennsylvania, Philadelphia, PA USA; 7 Laboratory of Biochemical Neuroendocrinology, Montreal Clinical Research Institute (IRCM), University of Montreal, Montreal, Quebec Canada

**Keywords:** Genetic engineering, Gene delivery

## Abstract

The viral delivery of base editors has been complicated by their size and by the limited packaging capacity of adeno-associated viruses (AAVs). Typically, dual-AAV approaches based on *trans*-splicing inteins have been used. Here we show that, compared with dual-AAV systems, AAVs with size-optimized genomes incorporating compact adenine base editors (ABEs) enable efficient editing in mice at similar or lower doses. Single-AAV-encoded ABEs retro-orbitally injected in mice led to editing efficiencies in liver (66%), heart (33%) and muscle (22%) tissues that were up to 2.5-fold those of dual-AAV ABE8e, and to a 93% knockdown (on average) of human PCSK9 and of mouse Pcsk9 and Angptl3 in circulation, concomitant with substantial reductions of plasma cholesterol and triglycerides. Moreover, three size-minimized ABE8e variants, each compatible with single-AAV delivery, collectively offer compatibility with protospacer-adjacent motifs for editing approximately 82% of the adenines in the human genome. ABEs encoded within single AAVs will facilitate research and therapeutic applications of base editing by simplifying AAV production and characterization, and by reducing the dose required for the desired level of editing.

## Abstract (web summary)

Adeno-associated viruses with size-optimized genomes encoding compact adenine base editors enable therapeutic base editing in mice at low doses and high editing efficiencies.

## Main

Gene editing offers the clinically validated potential to treat a wide variety of genetic disorders for which few therapeutic options are available. Because the study and treatment of most genetic disorders through gene editing require editing in vivo, clinically relevant methods that mediate the efficient delivery of precision gene editing agents into tissues in mammals^1,2^ continue to play a critical role in advancing the field.

Adeno-associated viruses (AAVs) have been used to deliver genes encoding many therapeutic proteins in animal models of human disease^3,4^, in clinical trials^5^ and in US Food and Drug Administration-approved drugs^6,7^. AAVs have become a popular in vivo delivery method due to its clinical validation, its ability to target a variety of clinically relevant tissues, and its relatively well-understood and favourable safety profile. Base editors^8,9^ efficiently install targeted transition mutations in a variety of therapeutically relevant cell types in vitro and in animal models of human genetic diseases^1,10^. Unlike nuclease-mediated gene editing, base editing does not require double-strand DNA breaks and therefore generates a minimum of unwanted indel byproducts, chromosomal translocations^11^, chromosomal aneuploidy^12^, large deletions^13,14^, p53 activation^15,16^ and chromothripsis^17^.

Base editors, which recently entered clinical trials, are generally too large to fit into a single AAV, which carry a cargo size limit of ~4.7 kb not including the inverted terminal repeats (ITRs)^18,19^. In addition to the base editor itself, AAVs that deliver base editors must also include the guide RNA, promoters driving base editor and single guide RNA expression, and *cis*-regulatory elements. Our group^20,21^ and others^22–26^ previously used AAVs to deliver base editors by dividing the base editor into two halves, each fused to a small *trans*-splicing intein^27^ or each expressed as messenger RNAs that undergo *trans*-splicing^28^. Although dual-AAV delivery of base editors has supported therapeutic levels of editing including in mouse models of human disease^21,22,25,29^, the development of a single-AAV base-editing system would further increase potential impact by simplifying the application, characterization and manufacturing of the base editor agent, and potentially increasing editing efficiency by obviating the need for simultaneous transduction of multiple AAVs. An ideal single-AAV base-editing system would also minimize the required dose of AAV, an important advance since clinical applications of AAVs are often constrained by dose-limiting toxicity^30^.

Adenine base editors (ABEs) are a particularly useful class of editing agents because they install A·T → G·C conversions that correct approximately half of all known pathogenic single-nucleotide polymorphisms^9^. Phage-assisted continuous evolution (PACE) of ABE7.10, the original adenine base editor, recently yielded TadA-8e, a deoxyadenosine deaminase with increased activity and broadened compatibility with Cas domains other than SpCas9^31^. ABEs containing only a single TadA deaminase domain, rather than a single-chain dimer, retain activity^31,32^, further reducing editor size. A single-AAV ABE that uses the ABE7.10 TadA monomer inserted into SaCas9 was recently described but showed only <0.25% editing in vitro and was not assessed in vivo^33^. Moreover, whereas SaCas9 is small enough to provide a single-AAV-compatible base editor, its utility is greatly limited by the rarity of its NNGRRT protospacer adjacent motif (PAM). Since base editing requires the presence of a suitable PAM to place the target nucleotide within the editing window, ABEs that collectively offer broad PAM compatibility along with simple and efficient in vivo delivery would advance in vivo applications of base editing. While this work was in revision, Sontheimer and co-workers reported single-AAV delivery of Nme2ABE8e in vivo, with optimizations that supported up to 34% editing at the Rosa26 locus in liver^34^. While this report demonstrates single-AAV in vivo delivery of ABEs with higher efficiency than those previously reported, increased editing efficiencies and broader PAM compatibility are needed to increase the therapeutic relevance of single-AAV base editing.

In this study, we constructed small, highly active ABE8e variants and identified minimal necessary *cis*-acting components on the AAV genome to develop highly efficient single-AAV vectors with broad in vivo targeting capability. We characterized ABE8e variants that use compact CjCas9, Nme2Cas9 and SauriCas9 targeting domains to develop a suite of single-AAV high-activity adenine base editors that collectively offer compatibility with a broad range of PAM sequences, including commonly occurring N_4_CC and N_2_GG PAMs, enabling base editing of ~82% of adenines in the human genome in principle. Finally, we assessed the performance of single-AAV ABEs in mice by using them to install base edits associated with decreased cardiovascular disease risk, resulting in efficient editing (averaging 50%) of human *PCSK9*, mouse *Pcsk9* and mouse *Angptl3* in bulk liver at a range of clinically relevant doses with concomitant substantial reduction in circulating target protein, total cholesterol and triglycerides. Our findings advance the therapeutic potential of base editing, establish the benefits of single-AAV base editor constructs and provide a suite of single-AAV adenine base editors with broad collective targeting capability that support efficient in vivo base editing.

## Results

### Development of a size-minimized AAV backbone for ABE delivery

Starting with the small, robust editor SaABE8e (3.9 kb)^31^ and its PAM-variant SaKKH-ABE8e^31,35^, we set out to optimize the components of the AAV genome to yield size-minimized AAV-based delivery of adenine base editors in which the entire base editor, its guide RNA, and all necessary promoters and regulatory sequences are present in a single AAV (≤~5.0 kb, including ITRs). We first identified high-efficiency guide RNAs targeting *Pcsk9* to allow evaluation of in vivo genome editing (irrespective of protein knockdown) in N2A and 3T3 cells by transfecting plasmids encoding SaABE8e or SaKKH-ABE8e and corresponding sgRNAs with spacers targeting the endogenous *Pcsk9* gene. The editing efficiency of each sgRNA was analysed by targeted high-throughput DNA sequencing (HTS) (Supplementary Fig. 1). The most efficient guide RNA installed a W8R coding mutation in *Pcsk9* using SaABE8e (Supplementary Fig. 1).

To encode the full-length SaABE8e protein on a single-AAV genome, we first used the small ubiquitous promoter EFS (EF-1α short) and a terminator we previously validated to yield efficient split-intein base editor delivery: the gamma portion of woodchuck hepatitis virus post-transcriptional regulatory element (WPRE, gamma subunit W3) with bovine growth hormone (bGH) polyadenylation signal^36^. To simplify production when testing multiple AAV architectures, sgRNA targeting *Pcsk9* W8 was provided on a separate AAV.

**Fig. 1:**
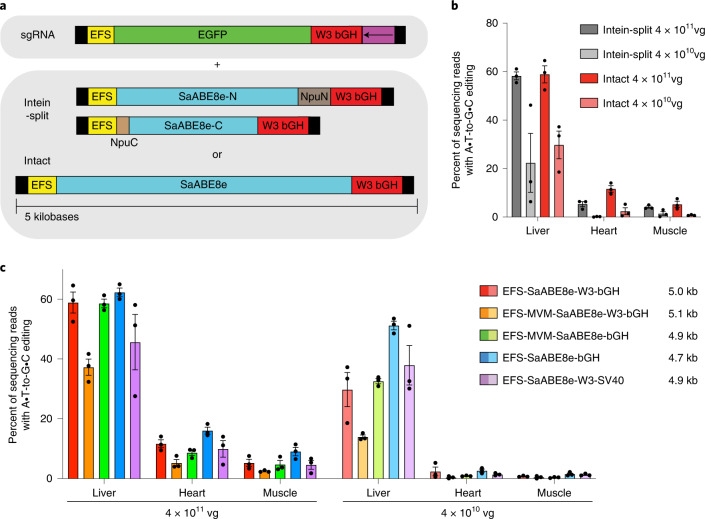
AAV constructs tested in vivo. **a**, sgRNA targeting *Pcsk9* W8 was delivered with EGFP in one AAV that was co-injected with either one or two additional AAVs encoding either an intact or intein-split SaABE8e, respectively. Thus a total of two AAVs were used to deliver the intact SaABE8e and sgRNA, and three AAVs were used to deliver the intein-split SaABE8e. Black boxes represent ITRs, EFS promoter is EF1a short, W3 is truncated WPRE, bGH is bovine growth hormone polyadenylation signal, the purple box is the U6 promoter-driven sgRNA cassette in the orientation indicated by the arrow, NpuN and NpuC split inteins from *Nostoc punctiforme* are shown in brown, and protein coding regions are indicated for EGFP and SaABE8e. **b**, In vivo editing efficiency from injection of AAV encoding intein-split and intact SaABE8e. The total dose of base editor AAV administered to each mouse is shown. **c**, Comparison of in vivo editing efficiency from injection of AAV9 encoding intact SaABE8e in five different AAV architectures when administered at the dose shown. In all cases, editor AAV dose was either 4 × 10^11^ vg or 4 × 10^10^ vg and sgRNA EGFP AAV dose was either 4 × 10^11^ vg or 4 × 10^10^ vg for a 1:1 ratio of base editor AAV to sgRNA AAV. The sizes of the delivered editor AAV constructs (including ITRs) are shown in the legend. C57BL/6J mice 6–7 weeks of age and 20–25 g were injected systemically by retro-orbital injection. Dots represent individual mice. Values and error bars represent mean ± s.e.m. of *n* = 3 different mice.

We first compared the in vivo editing activity of intact SaABE8e delivered on a second AAV to that of intein-split SaABE8e delivered on a second and third AAV using a previously validated intein-split site^22^ (Fig. 1a). To assess editing across multiple tissues, we systemically administered by retro-orbital injection a mixture of two or three AAV9 encoding (1) enhanced green fluorescent protein (EGFP) and sgRNA targeting *Pcsk9* and (2) either the intact AAV9 SaABE8e or the intein-split AAV9 SaABE8e (Fig. 1a) into 6–7-week-old wild-type C57BL/6J mice. We injected either a high total dose (8 × 10^11^ vg or 4 × 10^13^ vg kg^−1^) or low total dose (8 × 10^10^ vg or 4 × 10^12^ vg kg^−1^) of AAV consisting of a 1:1 mixture of sgRNA AAV to total base editor AAV. We purposefully chose moderate doses of AAV to avoid saturating editing efficiency and increase the likelihood of observing differences in editing outcomes between different ABE-AAV architectures. At three weeks post injection, we collected liver, heart and muscle for analysis by HTS.

Both intact and intein-split SaABE8e AAVs resulted in dose-dependent and tissue-dependent editing activity consistent with the tropism of AAV9 across collected tissues^37,38^, with liver showing the highest editing efficiencies, followed by heart, then skeletal muscle. Intact SaABE8e AAV yielded robust editing at both doses administered, reaching 59%, 12% and 5.3% editing of bulk liver, heart and skeletal muscle, respectively, comparable to or higher than the editing efficiencies achieved with intein-split SaABE8e in all tested tissues and doses (Fig. 1b). Decreasing the amount of sgRNA AAV to one half or one quarter of the amount of base editor AAV did not affect editing efficiency in the liver, but decreased editing efficiency by ~1.4-fold per 2-fold decrease in administered sgRNA AAV in both heart and muscle (Supplementary Fig. 2), indicating that the dose of guide RNA partially limits editing efficiency in extrahepatic tissues under these conditions.

Next, we sought to improve editing efficiency and minimize the size of ABE AAV by identifying minimal necessary elements on the AAV genome. We designed and compared five AAV genome architectures for delivery of SaABE8e to assess the impact of modifying the EFS promoter by adding a minimal minute virus of mice (MVM) intron^39^, modifying the terminator by removing the truncated WPRE gamma subunit W3, or replacing the bGH polyadenylation signal with an SV40 late polyadenylation signal. We designed and produced the following AAV expression cassettes: (1) EFS-SaABE8e-W3bGH, (2) EFS-MVM-SaABE8e-W3bGH, (3) EFS-MVM-SaABE8e-bGH, (4) EFS-SaABE8e-bGH and (5) EFS-SaABE8e-W3-SV40. Each of the five AAV candidates was administered to 6–7-week-old wild-type C57BL/6J mice by retro-orbital injection at a high (4 × 10^11^ vg editor AAV plus 4 × 10^11^ vg sgRNA AAV) or low (4 × 10^10^ vg editor AAV plus 4 × 10^10^ vg sgRNA AAV) dose (Fig. 1c).

At 3 weeks post injection, we collected liver, heart and skeletal muscle, and analysed each tissue by HTS. Editing efficiencies followed a consistent pattern among architectures, with the highest efficiency construct of EFS promoter driving SaABE8e expression and a bGH polyadenylation signal without W3 (EFS-SaABE8e-bGH) outperforming the other architectures across all assessed doses and tissues. These data demonstrate that the *cis*-acting W3 element is not necessary for sufficient expression of SaABE8e from the EFS promoter in these tissues and cell types, and highlights the importance of assessing AAV elements in the context of a specific editing application.

### Development of a single-AAV adenine base editing system

**Fig. 2:**
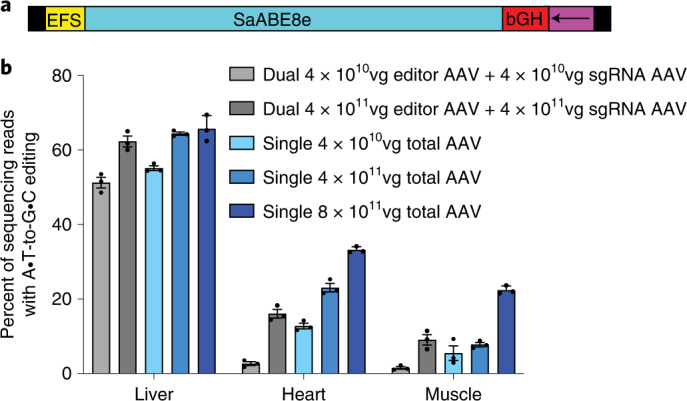
Development and characterization of a single-AAV SaABE8e. **a**, Schematic of the single-AAV SaABE8e genome (5,064 bp including ITRs). Arrow indicates direction of the U6 sgRNA cassette. **b**, Comparison of dual to single SaABE8e. Base editing activity of either a dual (SaABE8e with intact editor on one genome and sgRNA and EGFP on a second genome) or a single (SaABE8e and guide RNA all in one) AAV, both installing *Pcsk9* W8R packaged in AAV9 with matched promoter and terminator. Base editor AAVs were administered at the dose indicated in the legend (dual AAVs were delivered at the dose indicated for editor AAV and sgRNA AAV, whereas single AAVs were delivered at the dose indicated) to C57BL/6J mice (6–8 weeks old, weighing 20–25 g) via retro-orbital injection and tissues were collected at 3 weeks post injection, then analysed by HTS. Dots represent individual mice and error bars represent mean ± s.e.m. of *n* = 3 different mice.

The space gained by removal of W3 (250 bp) allowed the addition of an sgRNA expression cassette on the AAV genome, thereby enabling a single AAV with both ABE and guide RNA expression cassettes (Fig. 2a). We inserted the U6 sgRNA cassette proximal to the 3’ ITR, as we previously found this orientation to enhance base editing activity in intein-split BE AAVs^36^. This single-AAV9 SaABE8e was injected retro-orbitally into 6–8-week-old C57BL/6J mice at a dose of 4 × 10^11^ vg or 4 × 10^10^ vg, matching the dose of base editor AAV used in previous experiments, and corresponding to half of the previously used total AAV dose since the sgRNA was now expressed from the same AAV as the base editor. Single-AAV SaABE8e performed similarly at half the total AAV dose to intact SaABE8e and sgRNA expressed from two different AAVs in the liver at both the high and low doses. Single-AAV SaABE8e yielded 64% and 55% editing of bulk liver at high and low dose, respectively (Fig. 2b), similar to the editing achieved with dual-AAV SaABE8e at high and low doses. Single-AAV SaABE8e also resulted in 23% and 13% editing of bulk heart tissue at the high and low dose, respectively, corresponding to 1.4-fold and 4.8-fold higher editing efficiency compared with dual-AAV ABE (Fig. 2b, *P* = 0.038 and *P* = 0.0012, respectively, by unpaired *t*-test). Single-AAV SaABE8e yielded comparable editing to dual-AAV SaABE8e at the high and low doses in skeletal muscle compared with dual-AAV treatment, yielding 7.8% and 5.5% editing at high and low doses, respectively (Fig. 2b).

Next we assessed the single-AAV ABE construct at a dose of 8 × 10^11^ vg per mouse, equal in total AAV dose per mouse to that of the high-dose experiments described above requiring two AAVs. We observed further improvements in editing compared with the lower doses, especially in heart and muscle, which were now edited with 33% and 22% average efficiency, respectively. This level of editing corresponds to a 2.1-fold and 2.5-fold increase in editing in heart and muscle, respectively, compared with the highest observed level of base editing from dual-AAV SaABE8e with editor and sgRNA delivered on separate AAVs (Fig. 2b, *P* = 0.00048 and *P* = 0.0020, respectively, by unpaired *t*-test) at the same total dose of AAV. The relatively wide editing window of SaABE8e is maintained in vivo and indels remain low in each tissue (Supplementary Fig. 3).

**Extended Data Fig. 1:**
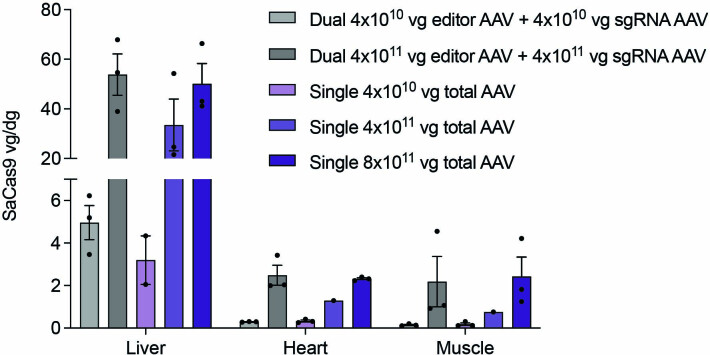
Quantification of AAV genomes from tissue. Quantification of AAV genomes from tissue encoding SaABE8e dual AAV (SaABE8e with intact editor on one genome and sgRNA and EGFP on a second genome) or single AAV (SaABE8e and guide RNA all-in-one), both installing *Pcsk9* W8R packaged in AAV9. Editors were packaged in AAV9 and administered by retro-orbital injection at 6–8 weeks of age at the dose indicated in the legend (dual AAVs were delivered at the dose indicated in the legend of editor AAV and sgRNA AAV, while single AAVs were delivered at the total dose indicated). Tissues were harvested 3 weeks post injection then editor AAV genomes were quantified by ddPCR using SaCas9 primers and probe, normalized to *Gapdh*. Dots represent individual mice (n = 1–3) and error bars show s.e.m.

We also quantified delivered editor AAV genomes from tissue by digital droplet PCR (ddPCR) and found that editing efficiencies correlate with the quantity of delivered genomes in each tissue (Extended Data Fig. 1), with liver being much more amenable to transduction with AAV9 than heart and muscle, consistent with previous analysis of the biodistribution of AAV9^40^. We found that tissues with largest differences in editing between single- and dual-AAV strategies were less efficiently transduced than liver. Heart and muscle were similarly transduced, which may indicate that the EFS promoter is more active in heart or may reflect effects of tissue heterogeneity.

These levels of base editing achieved in the liver, heart and muscle from single-AAV ABE injection would be sufficient to offer therapeutic benefit for many disorders^21,41–43^. These data also represent some of the highest reported somatic cell in vivo base editing in these tissues at clinically relevant doses^21,26,36,44,45^ of ≤10^14^ vg kg^−1^. Together, these results demonstrate that this engineered single-AAV ABE architecture mediates robust genome editing in vivo and highlights the benefits of single-AAV systems over dual-AAV methods, especially in less well-transduced tissues or when using lower total doses of AAV.

### Development of a suite of size-minimized ABEs with broad collective PAM compatibility

**Fig. 3:**
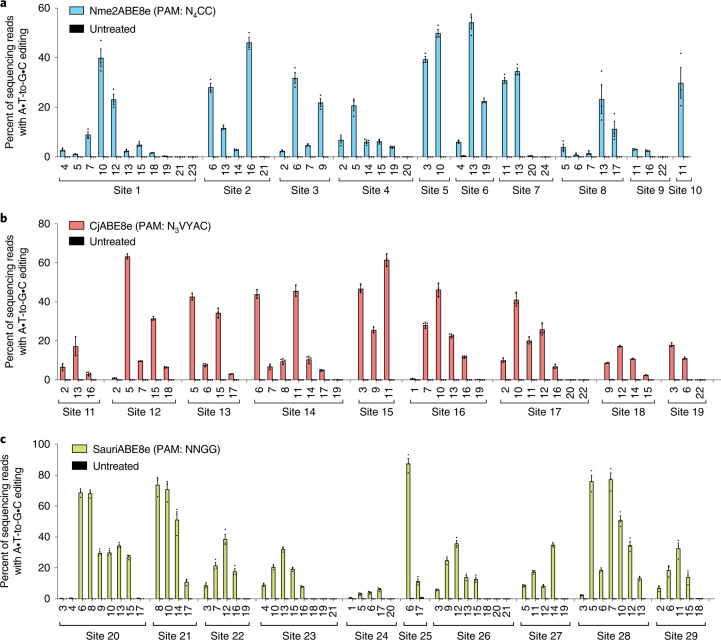
Characterization of size-minimized-ABEs in HEK293T cells. **a**–**c**, Nme2ABE8e (**a**), CjABE8e (**b**) and SauriABE8e (**c**) base editing of each target adenine within the protospacer. Target sites are indicated, with sequences of each target protospacer and PAM listed in Supplementary Table 1. Target adenines are numbered with respect to a standard protospacer length for each editor (22 nt for CjABE8e, 24 nt for Nme2ABE8e and 21 nt for SauriABE8e). Dots represent values and error bars represent mean ± s.e.m. of *n* = 3 biological replicates.

To broaden the targeting scope of single-AAV ABEs beyond that of SaCas9 (3.16 kb; PAM, NNGRRT) or engineered variants such as SaKKH^35^ (3.16 kb; PAM, N_3_RRT), we profiled the editing activity of ABE8e that used the nickase forms of the small-Cas orthologues Nme2Cas9^46,47^ (3.24 kb; PAM, N_4_CC), CjCas9^48–50^ (2.95 kb; PAM, N_3_VRYAC) and SauriCas9^51^ (3.18 kb; PAM, N_2_GG). To profile the activity of these size-reduced ABEs across multiple loci, plasmids encoding each editor and a corresponding sgRNA targeting a PAM-matched site were transfected into HEK293T cells. Three days later, the cells were analysed by targeted high-throughput DNA sequencing (Fig. 3a–c).

**Fig. 4:**
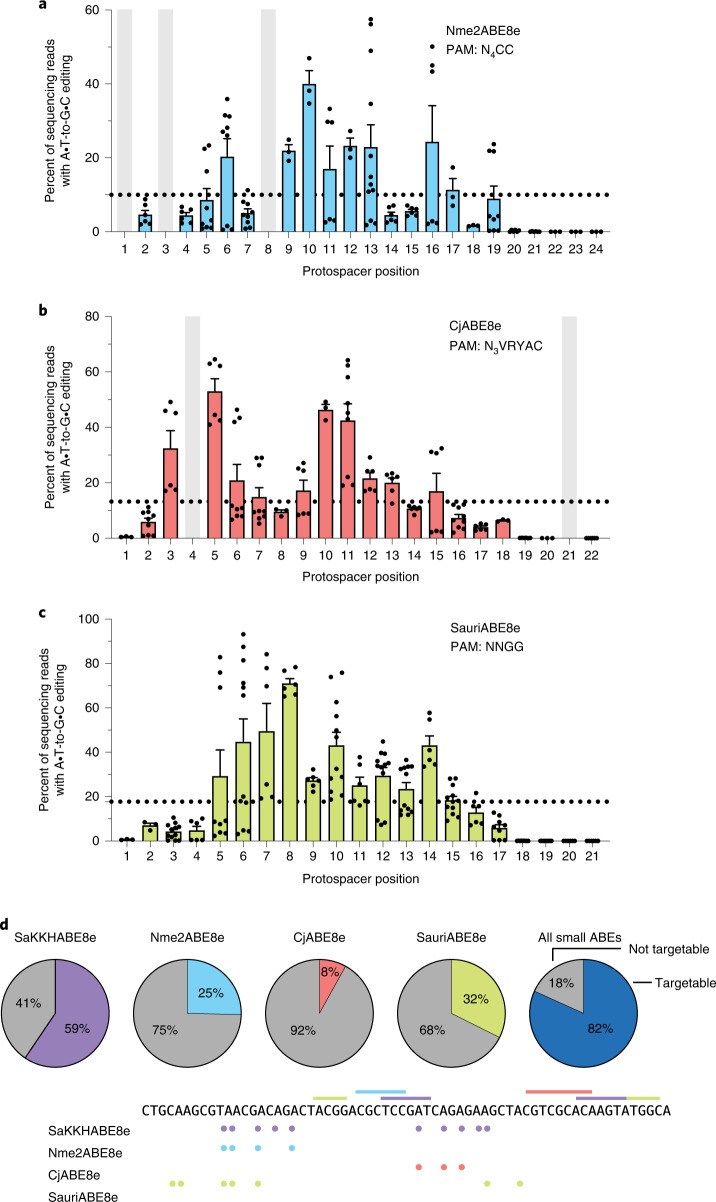
Base editing activity window summary of size-minimized ABEs in HEK293T cells. **a**–**c**, Base editing activity windows from 8–9 genomic target sites for Nme2ABE8e (**a**), CjABE8e (**b**) and SauriABE8e (**c**). Positions that were not present in any tested site are shaded grey. Target adenines are numbered with respect to a standard protospacer length for each editor (24 nt for Nme2ABE8e, 22 nt for CjABE8e and 21 nt for SauriABE8e). Dots represent values and error bars represent mean ± s.e.m. of *n* = 3 biological replicates for each site, with each position representing 1–3 genomic sites. The dotted line corresponds to 25% of mean peak activity for each base editor and defines the activity threshold considered within the editing window. Individual sites that contained three or more adenines within a protospacer were included in this analysis. Sites with only one or two adenines within the protospacer were excluded from summary analysis, but data from all sites analysed are shown in Fig. 3. **d**, The percentage of genomic adenines in the hg38 human reference genome targetable by size-minimized ABEs, either independently or collectively. An example showing a representative portion of the human genome targetable by size-minimized ABEs is shown, with targetable PAMs denoted with coloured lines and targetable adenines denoted with coloured dots.

All three of the tested small ABEs supported efficient base editing in HEK293T cells, with peak efficiencies at each target site generally ranging from 40–70%. Consistent with previous studies on base editors composed of smaller Cas variants^31,52,53^, Nme2ABE8e, CjABE8e and SauriABE8e all exhibited broader base editing windows compared with SpCas9-based ABE8e. For Nme2ABE8e, the editing window spans much of the distal half of the 24 nt protospacer (position 2–19, counting the PAM as positions 25–30), with optimal editing occurring between positions 6 and 17 (Figs. 3a and 4a). For CjABE8, the smallest of the three small-Cas variants tested, the window was even larger, spanning positions 2–18, counting the PAM as positions 24–31, with optimal editing occurring between positions 3 and 15 (Figs. 3b and 4b). CjABE8e also appeared to be more sensitive to the context preferences of the fused deaminase than the other tested ABEs, with editing efficiency varying substantially depending on the nucleobase 5’ of the target adenine (YA » RA). The editing window of SauriCas9-ABE8e typically ranges from protospacer positions 3–16 (counting the PAM as positions 22–25), with optimal editing occurring between positions 5–15 (Figs. 3c and 4c), which resembles the wide editing window enabled by the related SaCas9. Notably, SauriCas9’s broad PAM compatibility (3’ NNGG) in principle allows access to virtually all previously characterized SpCas9 targets (3’ NGG PAM), but now with single-AAV base editors.

To assess the collective targeting scope of this suite of four small ABE8e variants (SaKKHABE8e, Nme2ABE8e, CjABE8e and SauriABE8e), we determined the number of adenines in the entire hg38 human reference genome that are targetable in principle by at least one of these variants. We analysed the surrounding sequence context of each adenine for the presence of a small-ABE8e-targetable PAM that would place each adenine within an appropriate base editing window. This analysis revealed that 82% of all adenines in the human genome are theoretically targetable by at least one of these four small ABE8e variants (Fig. 4d). These data suggest that the single-AAV ABE system can potentially target the vast majority of adenines across the genome, although bystander editing in most cases will result in additional mutations, approximately half of which will be non-silent^54^.

### Adenine base editing of Pcsk9 and Angptl3 with small-Cas ABEs in cultured cells

To test the in vivo therapeutic potential of the single-AAV ABE system, we installed in mice mutations that are associated with decreased cardiovascular disease risk in humans^55–57^ by precisely knocking down Pcsk9 or Angptl3 protein levels. Knockdown of these proteins reduces levels of serum biomarkers including circulating protein and total cholesterol, as well as triglycerides when knocking down Angptl3, facilitating robust functional assessment of editing efficiency. We used SaABE8e, SaKKH-ABE8e and the newly designed SauriABE8e to disrupt start codons, splice donors and splice acceptors^58^ to precisely block production of the targeted protein without relying on double-strand breaks or indel formation.

**Extended Data Fig. 2:**
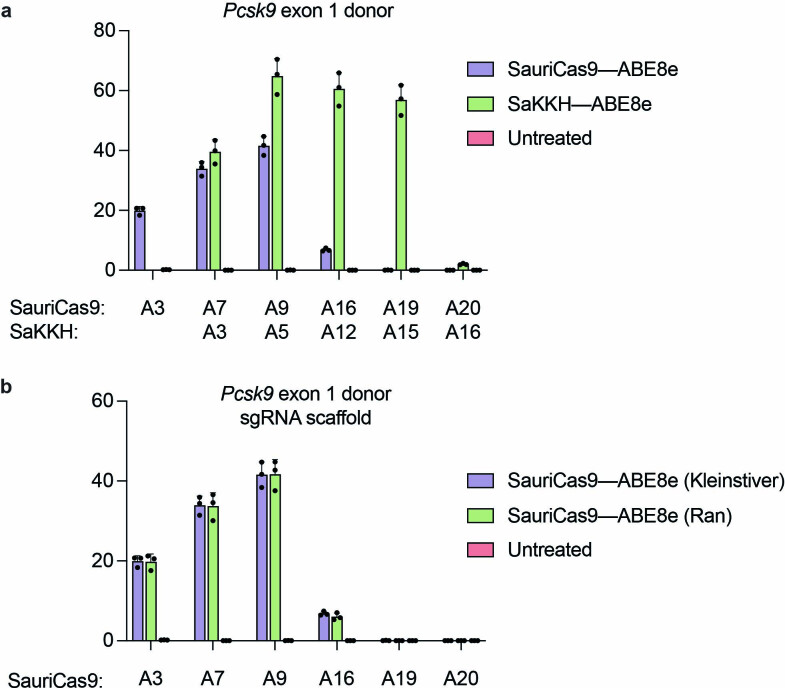
Editing in Neuro-2A cells with SauriABE8e at *Pcsk9* exon 1 splice donor. **a**, Targeting the mouse *Pcsk9* exon 1 splice donor with SauriABE8e and SaKKH-ABE8e in mouse Neuro-2A cells. The target adenine is A_9_ with respect to the Sauri protospacer, A_5_ with respect to SaKKH protospacer. **b**, A comparison of SaCas9 guide RNA scaffolds^1,2^ on editing activity at the mouse *Pcsk9* exon 1 splice donor. The SaCas9 sgRNA scaffold is used with the homologous SauriCas9 protein since the native sgRNA for SauriCas9 is not known.

We first designed and measured editing activity of guide RNAs expected to disrupt production of Pcsk9 or Angptl3 by transfection of plasmids encoding a size-reduced ABE8e and sgRNA targeting sites throughout human *PCSK9* in HEK293T cells (Supplementary Fig. 4a) or mouse *Pcsk9* and *Angptl3* in Neuro-2a cells (Supplementary Fig. 4b,c and Extended Data Fig. 2). Base editing efficiencies varied from undetectable to 89% as measured by deep sequencing of genomic DNA at the targeted loci. We advanced to in vivo experiments three highly efficient SaKKH-ABE8e guide RNAs targeting exon 1 splice donor of human *PCSK9*, and mouse *Pcsk9* and exon 6 splice donor of mouse *Angptl3*, as well as a SauriABE8e sgRNA targeting the exon 1 splice donor of mouse *Pcsk9*.

### Single-AAV adenine base editing of Pcsk9 and Angptl3 in mice

**Fig. 5:**
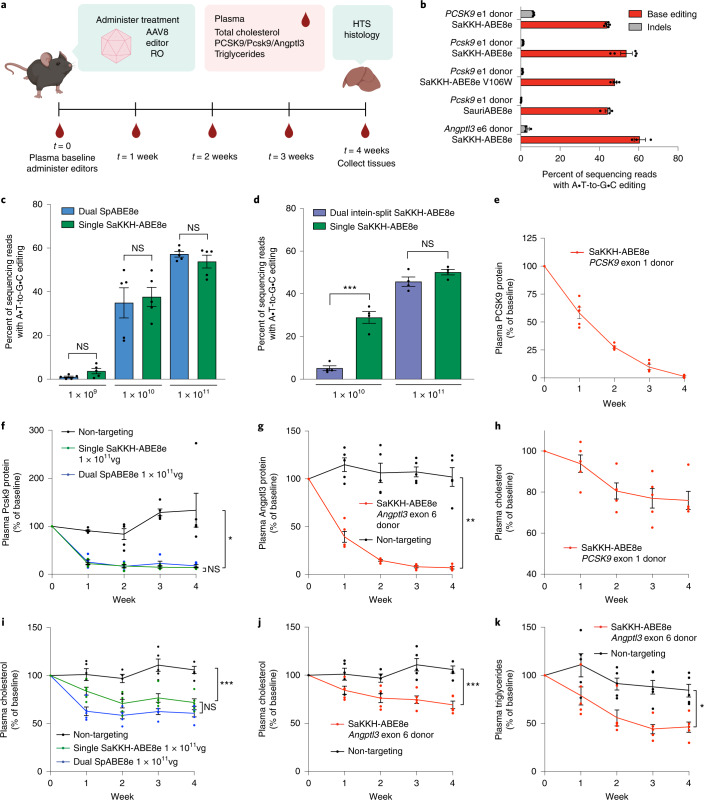
Assessment of genome editing and plasma lipids when targeting *PCSK9*, *Pcsk9* and *Angptl3* with single-AAV ABEs in vivo. **a**, Strategy for assessing base editing and plasma analytes in AAV-treated mice. **b**, Bulk liver editing efficiencies at human *PCSK9*, and mouse *Pcsk9* and *Angptl3* (*n* = 3–5 mice; each dot represents one mouse, error bars represent s.e.m.). Human *PCSK9* editing was performed using humanized *PCSK9* mice, whereas mouse *Pcsk9* and *Angptl3* editing was performed at the endogenous mouse loci of wild-type C57BL/6J mice. AAV was administered by retro-orbital (RO) injection at 6–8 weeks of age at a dose of 1 × 10^11^ vg per mouse. **c**, Dose-dependent base editing for dual SpABE8e and single SaKKH-ABE8e at mouse *Pcsk9* exon 1 splice donor. The total AAV dose administered is indicated below each set of bars in vg per mouse. The dual SpABE8e editing data are reported in another publication from our group^2^. Each dot represents a different mouse (*n* = 5). **d**, Direct comparison of editing efficiencies of dual-AAV8 intein-split SaKKH-ABE8e and single-AAV8 SaKKH-ABE8e targeting the *Pcsk9* exon 1 donor site in bulk liver at two doses. The total AAV dose administered is indicated below each set of bars in vg per mouse, ***P = 0.0004. Each dot represents a different mouse (*n* = 4). For **b**-**d**, liver tissue was harvested at four weeks post injection and analyzed by HTS. **e**, Plasma PCSK9 protein in humanized mice treated with 1 × 10^11^ vg single-AAV8 SaKKH-ABE8e. **f**, Plasma Pcsk9 protein in C57BL/6J mice treated with either 1 × 10^11^ vg single-AAV8 SaKKH-ABE8e, dual-AAV8 SpABE8e or non-targeting control, ***P = 0.0001. **g**, Plasma Angptl3 protein in C57BL/6J mice treated with 1 × 10^11^ vg single-AAV8 SaKKH-ABE8e or non-targeting control, **P = 0.0027. **h**, Plasma total cholesterol in humanized mice treated with 1 × 10^11^ vg single-AAV8 SaKKH-ABE8e. **i**, Plasma total cholesterol in C57BL/6J mice treated with either 1 × 10^11^ vg single-AAV8 SaKKH-ABE8e, dual-AAV8 SpABE8e or non-targeting control, ***P = 0.0007. **j**,**k**, Plasma total cholesterol (**j**, ***P = 0.0007) and plasma triglycerides (**k**, *P = 0.0118) in C57BL/6J mice treated with 1 × 10^11^ vg single-AAV8 SaKKH-ABE8e or non-targeting control. For **e**–**k**, dots represent individual mice and error bars represent s.e.m. of *n* = 5 different mice. Significance was calculated for **c** and **d** using two-way unpaired *t*-test. Significance for **f**, **g** and **i**–**k** was calculated using two-way repeated measures ANOVA with Tukey’s or Sidak multiple comparisons, as applicable, and is shown for the week 4 timepoint for all graphs except for **f**, in which week 3 significance is shown as week 4 protein levels did not reach statistical significance. In all instances, non-targeting control is dual-AAV8 SpABE7.10 with sgRNA targeting mouse *Dnmt1*, an unrelated site in the mouse genome, administered at the same timepoint, route and dose.

To assess in vivo editing activity with optimized sgRNAs targeting *PCSK9*, *Pcsk9* and *Angptl3* together with the corresponding size-minimized ABE8e variants, we prepared single-AAV ABEs in AAV8, a serotype that efficiently transduces murine hepatocytes^59^, and administered them to 6–8-week-old mice systemically via retro-orbital injection at a dose of 1 × 10^11^ vg per mouse (5 × 10^12^ vg kg^−1^) (Fig. 5a). We injected AAV8 encoding SaKKH-ABE8e and sgRNA targeting the exon 1 splice donor of human *PCSK9* into humanized mice containing the human *PCSK9* sequence^60^. Similarly, AAV8 encoding SaKKH-ABE8e, SaKKH-ABE8e V106W^61^, or SauriABE8e and editor-matched sgRNA targeting exon 1 splice donor of mouse *Pcsk9* or SaKKH-ABE8e and sgRNA targeting the exon 6 splice donor of mouse *Angptl3* were injected to wild-type C57BL/6J mice. After 4 weeks, bulk liver tissue was analysed by HTS. These treatments achieved 44%, 54%, 47%, 46% and 61% base editing of bulk liver tissue for human *PCSK9* using SaKKH-ABE8e, mouse *Pcsk9* using SaKKH-ABE8e, mouse *Pcsk9* using SaKKH-ABE8e V106W, mouse *Pcsk9* using SauriABE8e, and mouse *Angptl3* using SaKKH-ABE8e, respectively (Fig. 5b). The relative editing efficiency of each target in cultured HEK293T and N2A cells (Supplementary Fig. 4) paralleled relative editing efficiencies in vivo (Fig. 5b). SaKKH-ABE8e V106W, which uses a mutant of evolved TadA-8e deaminase that reduces guide-independent DNA and mRNA off-target editing^61^, maintained high editing efficiency in vivo (Fig. 5b). These single-AAV in vivo base editing efficiencies approach those of state-of-the-art lipid nanoparticle (LNP)-mediated ABE mRNA liver delivery methods targeting *Pcsk9* and *Angptl3* recently reported in preclinical studies in mice^44,45,62^.

We also compared the editing activity of single-AAV8 SaKKH-ABE8e (1 × 10^11^ vg) targeting the *Pcsk9* exon 1 splice donor to the previously optimized^36^ dual-AAV split-intein SpABE8e architecture paired with an SpCas9 sgRNA validated to efficiently edit and knockdown Pcsk9 in vivo^44,45^ by targeting the same splice donor. We administered single-AAV8 SaKKH-ABE8e and dual-AAV8 SpABE8e at the same total dose per mouse to 6–8-week-old C57BL/6J mice at 3 doses (1 × 10^11^ vg, 1 × 10^10^ vg or 1 × 10^9^ vg total AAV per mouse) and measured disruption of *Pcsk9* exon 1 spice donor in liver at 4 weeks after administration, by HTS. The maximum vg kg^−1^ dose used (5 × 10^12^ vg kg^−1^ for a 20 g mouse) is comparable to or lower than those used in gene therapy non-human primate studies and human clinical trials^6,63^. We observed that the single- and dual-AAV ABE systems performed similarly at each dose, with the single-AAV yielding 54%, 38% and 3.7% average editing in liver at a dose of 1 × 10^11^ vg, 1 × 10^10^ vg and 1 × 10^9^ vg, respectively (Fig. 5c), and editing via dual-AAV SpABE8e in liver at the same dose of AAV averaging 57%, 35% and 1.0%, respectively (no significant difference at any dose by unpaired *t*-test). These results collectively show that single-AAV ABE performs comparably to the highly active previously optimized dual-AAV SpABE8e at a range of doses at a therapeutically relevant locus^36^.

To investigate the modest apparent editing efficiency improvement of the single-AAV system compared with split SpABE8e in liver, we directly compared single-AAV8 SaKKH-ABE8e and dual-AAV8 intein-split SaKKH-ABE8e with matched promoter and polyA by systemic retro-orbital delivery to 6–8-week-old C57BL/6 mice at two doses (Fig. 5d). Single- and dual-AAV intein-split SaKKH-ABE8e show similar editing at the high dose of 1 × 10^11^ vg (50% and 46%, respectively, not significant by unpaired *t*-test) but markedly lowered activity for the dual-AAV intein-split SaKKH-ABE8e compared with single-AAV SaKKH-ABE8e at a dose of 1 × 0^10^ vg (5.2% versus 29%, respectively, *P* = 0.0004). These data indicate that single-AAV SaKKH-ABE8e may perform similarly to optimized dual-AAV SpABE8e under conditions in which AAV transduction is already at or near saturation, perhaps because the increased activity of SpABE8e and/or increased activity afforded by the *cis*-regulatory elements that can be included with the extra space on two AAV genomes can overcome limitations of intein-splitting and dual transduction. For applications in which AAV transduction is well below saturation, however, single-AAV delivery can result in substantial editing efficiency improvements. We also assessed the integrity of AAV genomes packaged in single-AAV ABEs, by alkaline gel electrophoresis of single-AAV ABEs as well as smaller intein-split dual-AAV ABEs (Supplementary Fig. 5) and found that single-AAV ABEs package full-length and truncated genomic species, consistent with their size being at the AAV packaging limit^19^.

### Reduction in circulating protein and lipids upon editing of Pcsk9 and Angptl3

To assess whether the efficient editing observed in the liver can translate into efficient target gene knockdown and concomitant reduction in circulating lipid levels, we serially bled editor-treated mice and measured plasma levels of the targeted protein and total cholesterol. A non-targeting control of dual-AAV ABE7.10 targeting *Dnmt1*, an edit not expected to affect cholesterol or lipid metabolism, was included for comparison (Supplementary Fig. 6). We observed nearly complete protein knockdown in all experimental conditions at a dose of 1 × 10^11^ vg (5 × 10^12^ vg kg^−1^) single-AAV SaABE8e or SaKKH-ABE8e by 4 weeks, with most knockdown evident by 2 weeks (Fig. 5e,f,g, and Supplementary Figs. 7a,c and 8a,c). On average, single-AAV ABE treatment at this dose resulted in 99%, 91% and 94% knockdown of human PCSK9, mouse Pcsk9 and mouse Angptl3 protein levels, respectively, compared with control animals treated with AAV encoding the *Dnmt1-*targeting guide RNA, again matching the knockdown of these proteins achieved by state-of-the-art LNP-mediated ABE mRNA delivery to the liver^45,62^. This high level of protein knockdown is also consistent with observed editing levels (Fig. 5b) since the hepatocytic tropism of AAV8^64^ and the fact that hepatocytes constitute roughly 70% of the murine liver^65^ imply that 44–61% bulk liver editing corresponds to ~60–85% editing of hepatocytes.

Protein knockdown also resulted in decreased circulating cholesterol in all ABE-treated mice (Fig. 5h–j, and Supplementary Figs. 7b,d and 8b,d). Plasma total cholesterol in human *PCSK9*-targeted mice decreased by 24% from baseline levels to 45 mg dl^−1^ after 4 weeks. At the highest AAV dose in *Pcsk9*-targeted mice, plasma cholesterol was lowered by an average of 25% compared with age-matched non-targeting controls to 53 mg dl^−1^ after 4 weeks (Fig. 5i), similar to the degree of cholesterol lowering observed in liver-specific *Pcsk9* knockout mice^66^. For *Angptl3*-targeted mice, we observed a 38% decrease in plasma cholesterol compared with age-matched non-targeting controls to 44 mg dl^−1^. These results demonstrate substantial lowering of cholesterol using single-AAV ABEs.

We assessed the dose-dependence of AAV dose and editing on circulating Pcsk9 and total cholesterol. Knockdown of mouse Pcsk9 and decrease in total plasma cholesterol were dose-dependent and closely reflected the level of editing observed at each dose (Supplementary Fig. 7). Dual SpABE8e, which effected editing to similar levels as single-AAV ABE8e, also resulted in decreased plasma cholesterol in a dose-dependent manner (Supplementary Fig. 7). For both single- and dual-AAV ABEs targeting mouse Pcsk9, cholesterol and protein knockdown correlated closely with editing percentage, regardless of editor type administered.

We also measured plasma triglycerides in *Angptl3*-targeted mice, as loss-of-function alleles of *Angptl3* are known to reduce levels of both cholesterol and triglycerides^67^. In the *Angptl3-*targeted mice, we observed a 45% decrease in circulating triglycerides compared with non-targeting control to 25 mg dl^−1^ after 4 weeks (Fig. 5k and Supplementary Fig. 8e). The editing and reduction of circulating Angptl3, cholesterol and triglycerides achieved here with single-AAV ABE is, to our knowledge, the highest thus far reported upon targeted genome editing to knockdown *Angptl3*^62,68^. Together, these results demonstrate robust base editing at multiple therapeutically relevant loci achieved with single-AAV ABEs, resulting in strong effects on target protein level and metabolic changes in adult mice.

**Extended Data Fig. 3:**
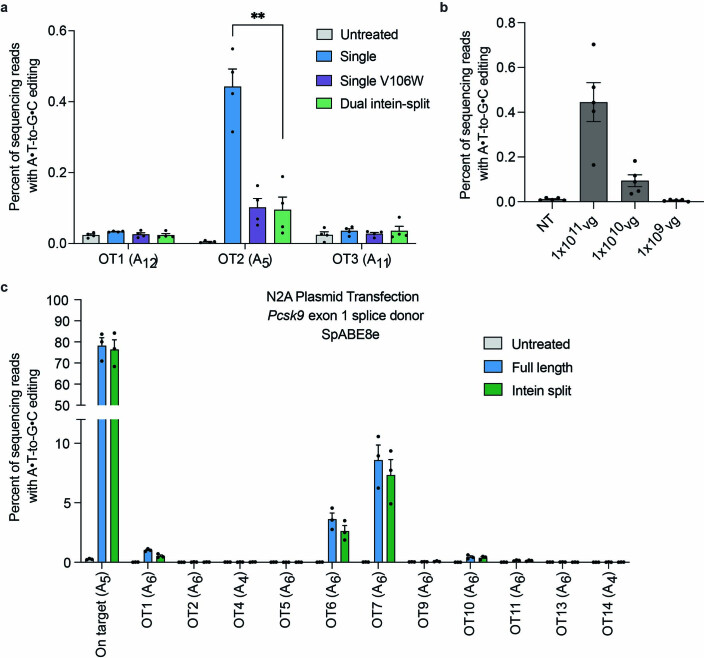
Guide-dependent off-target DNA editing *in vivo* and in cell culture. **a**, Liver tissue from single-AAV ABE treated mice. The top three predicted off-target sites for SaKKH-ABE8e targeting *Pcsk9* exon 1 splice donor were sequenced from liver tissue. **b**, The editing observed at OT2 is dose-dependent. **c**, On- and off-target sites were sequenced after plasmid transfection of N2A cells with sgRNA targeting *Pcsk9* exon 1 donor site and full-length or intein-split SpABE8e. Full-length and intein-split SpABE8e did not significantly differ in efficiency at on- or off-target edits by multiple unpaired t tests with Holm-Sidak method for correction for multiple comparisons. OT, off-target; NT, non-targeting dose-matched dual AAV8 ABE7.10 targeting *Dnmt1*, an unrelated gene.

**Extended Data Fig. 4:**
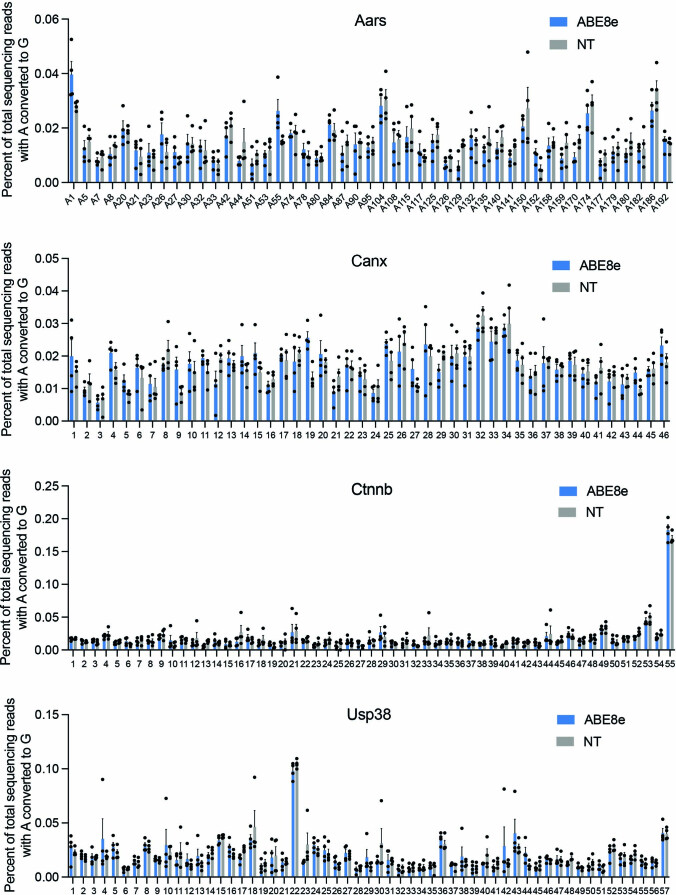
*In vivo* off-target mRNA editing. RNA was extracted from mouse livers treated with single-AAV8 SaKKH-ABE8e and untreated mouse livers, reverse transcribed, and cDNA amplicons from Aars, Canx, Ctnnb, and Usp38 mRNA were analyzed by HTS. Dots represent individual adenines across the sequenced amplicon (n = 3 mice).

Lastly, we assessed liver morphology and off-target editing in mice treated with single-AAV ABEs. We performed histology on livers from mice treated with single-AAV8 SaKKH-ABE8e and guide targeting PCSK9 exon 1 donor at 1 × 10^11^ vg 4 weeks after administration and did not find evident morphological changes compared to untreated mice (Supplementary Fig. 9). Although off-target editing in cell culture has been characterized for SaCas9-based genome editing agents, no off-target editing in tissues treated with SaCas9-based editing agents in vivo has been reported^22,69^. To assess single-AAV ABEs in vivo off-target editing, we sequenced the top three computationally predicted sites^70,71^ from liver tissue of C57BL/6J mice treated with 1 × 10^11^ vg single-AAV8 SaKKH-ABE8e, single-AAV8 SaKKH-ABE8e V106W, or dual-AAV8 intein-split SaKKH-ABE8e targeting mouse Pcsk9 exon 1 at 4 weeks after administration. We observed low but detectable (up to 0.45%) and dose-dependent editing at one off-target site in vivo (Extended Data Fig. 3a,b), suggesting the importance of considering off-target editing outcomes when using single-AAV ABEs, even though observed off-target editing was relatively rare at the sites examined. Off-target editing in vivo at this site was ameliorated by addition of the TadA V106W mutation, which has been reported to lower guide-independent DNA off-target editing and mRNA off-target editing^31,61^. Off-target editing in vivo was also ameliorated by delivery via dual-AAV intein-split SaKKH-ABE8e, which may be due to inherent lower overall activity of intein-linked ABE8e, or the lower dose of complete base editors, although we did not observe any significant difference between full-length or intein-split SpABE8e off-target editing in N2A cells by plasmid transfection in vitro (Extended Data Fig. 3c). Finally, we assessed in vivo off-target mRNA editing in single-AAV8 SaKKH-ABE8e-treated mice by analysing complementary DNA amplicons of mouse homologues of demonstrated ABE mRNA off-target human transcripts^61^, some containing partial TadA recognition sequences, and observed no off-target mRNA editing compared with untreated mice (Extended Data Fig. 4). These results suggest that single-AAV ABEs maintain low levels of off-target DNA and RNA editing in vivo for the guide RNA tested, and that deaminase mutations can further minimize off-target editing in vivo.

## Discussion

By minimizing the size of adenine base editors and AAV components, we developed a suite of single-AAV adenine base editor systems that support robust editing in vivo and have broad targeting capability due to their collective PAM compatibility. Single-AAV ABEs supported base editing efficiencies up to 66%, 33% and 22% editing in liver, heart and muscle, respectively, and outperformed dual-AAV approaches especially when tissue type or AAV dose prevented saturating levels of transduction. The largest editing efficiency increases compared with dose-matched dual-AAV were 2.1-fold in heart and 2.5-fold in skeletal muscle, potentially due to the relatively lower transduction efficiency in these tissues. These findings suggest that a single-AAV system may be especially preferable when targeting non-liver tissues, or when toxicity limits AAV dosage (Fig. 2b).

Single-AAV ABEs can be packaged in multiple serotypes (in this study, AAV8 and AAV9), which facilitates editing in a variety of tissues and cell types outside the liver, or with alternate administration routes. Even for base editing in the liver, the organ for which LNP-mediated mRNA delivery is the most potent, single-AAV ABEs resulted in editing efficiencies, target protein knockdown and desired phenotypic changes comparable to state-of-the-art preclinical LNP-mediated mRNA delivery efforts^44,45^. In organs, such as the heart, for which LNP-mediated delivery is not yet efficient, the single-AAV systems developed here may prove especially useful.

Single-AAV ABEs offer several potential advantages over dual-AAV approaches for clinical use: clinical-scale production of a single vector rather than two; increased potency, especially at lower doses; and reduced complexity from a simpler construct that obviates the need to use a *trans*-splicing intein. For these reasons, we anticipate that in vivo editing approaches compatible with single-AAV delivery may be more readily applied to large animal models and human therapeutics where systemic delivery is commonly used. Development of smaller promoters that provide sufficient expression of base editors will allow further minimization of the elements of single-AAV ABEs, which should facilitate clinical translation by increasing the proportion of full-length packaged AAV genomes.

Base editor delivery with single AAVs is currently limited to base editors that use small-Cas enzymes ≤~3.2 kb in gene size. Although we demonstrated activity of a variety of size-reduced ABEs that together cover a targetable genome similar to that targetable by SpCas9-ABE, single AAVs are still limited to A•T-to-G•C edits and have wide editing windows both in cell culture and in vivo that increase the possibility of bystander editing. We calculate that approximately 82% of genomic adenines can be edited using the suite of size-minimized ABEs described in this work. Whereas only a small fraction can be targeted without any bystander edits, for many applications, bystander editing may be acceptable, for example because: it results in silent or benign mutations, the target is in a non-coding regulatory sequence, or the application seeks to disrupt the function of a sequence. For those applications in which bystander edits are unacceptable, broad-window ABEs may not be suitable, highlighting the need to develop additional small base editors with diverse PAM compatibilities or sequence context requirements^35^ to maximize precise positioning of the base editor and minimize undesired bystander editing. Further work to expand single-AAV systems to include cytosine base editors, and to create single-AAV base editors with altered activity window locations^53,72^ would further broaden the applicability of single-AAV in vivo base editing.

The single-AAV ABE systems described here in general yield robust editing efficiencies in vivo, facilitating therapeutically relevant levels of editing in liver, heart and muscle tissue at moderate doses of AAV. Whereas AAVs allow the targeting of tissues currently inaccessible with technologies such as LNPs, toxicity associated with AAVs has recently become recognized in non-human primates (NHPs) and in clinical trials at high doses^73,74^. Animal studies have also indicated that AAV genomic integration may lead to hepatocellular carcinoma^75^, although a causal link between liver tumours and AAVs has not been established in humans treated with recombinant AAV vectors^76,77^. Additionally, since AAV-based ABE delivery does not benefit from the transience of delivery methods such as engineered virus-like particles^2^ or LNP-mediated mRNA delivery^44^, the off-target editing profile and immunogenicity of long-term expression of base editors will need to be evaluated for potential clinical applications. Whereas the therapeutic landscape of AAVs continues to be explored, these limitations suggest the potential safety advantages of highly potent editing agents that limit the amount of AAV required to achieve therapeutic target editing levels. Additionally, immune responses to gene editing agents delivered via AAVs have yet to be thoroughly characterized in large animal models. Early data indicate that stable genome editing using non-native nucleases expressed from AAV is achievable in non-human primates without major adverse effects, albeit with some loss of edited hepatocytes^78^. Methods for the inducible expression of editing agents^79^ and alternative delivery vectors^2,80,81^ could also further synergize with size-reduced base editors and mitigate potential side effects.

## Methods

### Molecular biology

Expression vectors for tissue culture were cloned using KLD, Gibson or USER assembly. sgRNA expression plasmids were cloned via KLD or Goldengate assembly to install protospacers as indicated in Supplementary Table 1. Plasmids were constructed via USER assembly or Gibson assembly of PCR-amplified fragments. Plasmids encoding recombinant AAV (rAAV) genomes were cloned by Gibson assembly of plasmid restriction fragments and PCR amplicons with Gibson-compatible overhangs. All plasmids for mammalian tissue culture experiments were purified using Plasmid Plus Maxiprep or Midiprep kits (Qiagen), ZymoPURE II Midiprep kit (Zymo Research) or PureYield Plasmid Miniprep kits (Promega). Key plasmids developed in this study will be available through Addgene.

### Culture and transfection of HEK293T and N2A cells

HEK293T cells (ATCC CRL-3216) and Neuro-2A cells (ATCC CCL-131) were maintained in Dulbecco’s modified Eagle’s medium (DMEM) plus GlutaMax (Thermo Fisher) supplemented with 10% (v/v) fetal bovine serum (FBS) at 37 °C with 5% CO_2_. At 16–24 h before transfection, HEK293T cells or N2A cells were seeded on 96-well plates (Corning) at 1.4 × 10^4^–2.0 × 10^4^ cells per well at >90% viability; for SauriABE transfections, HEK293T cells were seeded in 48-well plates (Corning) at 4.0 × 10^4^ cells per well at >90% viability. Cells in 96-well plates were transfected at approximately 70–85% confluency with 0.5 μl lipofectamine 2000 (Thermo Fisher), and 187.5 ng base editor plasmid and 37.5 ng sgRNA plasmid per well (180 ng editor and 60 ng sgRNA for SauriABE8e). Cells in 48-well plates were transfected with 1.5 μl lipofectamine 2000, with 750 ng editor and 250 μl sgRNA. Cells were cultured for 72 h after transfection, then media was removed, cells were washed with 1× PBS (Thermo Fisher), and genomic DNA was extracted by addition of 30–60 μl lysis buffer (10 mM Tris-HCl, pH 7.5–8.0, 0.05% SDS and 20 μg ml^−1^ proteinase K (New England Biolabs)) per well for 96-well plates and 150 μl per well for 48-well plates. Genomic DNA was stored temporarily at 4 °C or longer term at −20 °C until further use.

### High-throughput sequencing and data analysis

Genomic DNA was amplified by PCR using Phusion Hot Start II DNA polymerase or Phusion U Hot Start DNA polymerase with 0%–3% dimethylsulfoxide added. Barcodes for Illumina sequencing were added via a second PCR step, using 1 μl of the first PCR as a template. Total PCR cycles were kept to a minimum to avoid PCR bias. Barcoded PCR products were pooled according to amplicon, gel extracted (MinElute; Qiagen) and quantified by qPCR (KAPA; KK4824) or Qubit dsDNA HS assay kit (Thermo Fisher). Sequencing of pooled libraries was performed using Illumina MiSeq according to the manufacturer’s instructions. Primers for amplification of each locus from genomic DNA are compiled in Supplementary Table 1.

Sequencing reads were demultiplexed using MiSeq Reporter (Illumina). Alignment of amplicon sequences to reference sequence was performed using CRISPResso2^82^ with ‘discard_indel_reads’ on. For quantification of base editing, efficiency was calculated as percentage of (reads containing an A to G edit at given position without indels)/(number of total reads). Indels were calculated explicitly as (discarded reads)/(total aligned reads) × 100. Base editing at a given position was calculated explicitly as: (frequency of specified point mutation in non-discarded reads) × 100 × (100 – (indel reads))/100)).

### AAV production

AAV was produced as previously described^36^. HEK293T clone 17 cells (ATCC CRL-11268) were maintained in DMEM plus GlutaMax (Thermo Fisher) supplemented with 10% (v/v) FBS without antibiotic in 150 mm dishes (Thermo Fisher, 157150) at 37 °C with 5% CO_2_ and passaged every 2–3 d. Cells were split 1:3 the day before polyethyleneimine transfection with 5.7 μg AAV genome plasmid, 11.4 μg pHelper (Chlontech) and 22.8 μg rep-cap plasmid per plate. Media were exchanged for DMEM with 5% FBS the day after transfection. Four days after transfection, cells and media were collected using a rubber cell scraper (Corning), pelleted by centrifugation at 2,000 *g* for 10 min, resuspended in 500 µl hypertonic lysis buffer per plate (40 mM Tris base, 500 mM NaCl, 2 mM MgCl_2_ and 100 U ml^−1^ salt active nuclease (ArcticZymes, 70910–202)) and incubated at 37 °C for 1 h to lyse the cells. The media were decanted and combined with a 5× solution of 40% poly(ethylene glycol) 8,000 (PEG 8k; Sigma-Aldrich, 89510) in 2.5 M NaCl for a final concentration of 8% PEG/500 mM NaCl, incubated on ice for 2 h and then centrifuged at 3,200 *g* for 30 min. The pellet was resuspended in 500 μl of hypertonic lysis buffer per plate and added to the cell lysate. Crude lysates were either incubated at 4 °C overnight or taken immediately for ultracentrifugation.

Cell lysates were clarified by centrifugation at 2,000 *g* for 10 min and added to Beckman Quick-Seal tubes via 16-gauge 5” disposable needles (Air-Tite N165). A discontinuous iodixanol gradient was formed by sequentially floating layers: 9 ml 15% iodixanol in 500 mM NaCl and 1× PBS-MK (1× PBS plus 1 mM MgCl_2_ and 2.5 mM KCl), 6 ml 25% iodixanol in 1× PBS-MK, and 5 ml each of 40% and 60% iodixanol in 1× PBS-MK. Phenol red at a final concentration of 1 µg ml^−1^ was added to the 15, 25 and 60% layers to facilitate identification. Ultracentrifugation was performed using a Ti 70 rotor in a Sorvall wX+ ultracentrifuge (Thermo Fisher) at 58,000 r.p.m. for 2 h and 15 min at 18 °C. Immediately following centrifugation, 3 ml of solution was withdrawn from the 40–60% iodixanol interface via an 18-gauge needle. The solution was exchanged into cold PBS containing 0.001% F-68 using PES 100 kD MWCO columns (Thermo Fisher, Pierce 88533) and concentrated. The concentrated AAV solution was sterile filtered using a 0.22 µm filter, quantified by qPCR (AAVpro titration kit version 2; Clontech) and stored at 4 °C until use.

### Animals

All experiments in live animals were approved by the Broad Institute and University of Pennsylvania Institutional Animal Care and Use Committees and were consistent with local, state and federal regulations as applicable, including the National Institutes of Health Guide for the Care and Use of Laboratory Animals. C57BL/6J mice (000664) for use in experiments were purchased from The Jackson Laboratory. Humanized PCSK9 mice were reported previously^60^. All mice were housed in a room maintained on a 12 h light and dark cycle with ad libitum access to standard rodent diet and water except for 4 h fasts just before bleeds for plasma analysis.

### Retro-orbital injections

AAV was diluted into 100 µl of sterile 0.9% NaCl USP (Fresenius Kabi, 918610) before injection. Anaesthesia was induced with 2–4% isoflurane. Following induction, as measured by unresponsiveness to bilateral toe pinch, the right eye was protruded by gentle pressure on the skin, and an insulin syringe was advanced, with the bevel facing away from the eye, into the retrobulbar sinus where AAV solution was slowly injected. One drop of proparacaine hydrochloride ophthalmic solution (Patterson Veterinary, 07–885–9765) was then applied to the eye as an analgesic. At collection, mice were euthanized by carbon dioxide asphyxiation. Genomic DNA was purified from minced tissue using gDNAdvance kit (Beckman Coulter, A48705) according to the manufacturer’s instructions and used as template for high-throughput sequencing. RNA was purified from 30 mg of snap-frozen liver tissue with RNeasy Plus mini kit (Qiagen 74134) according to the manufacturer’s instructions, then reverse transcribed to cDNA using SuperScript III first-strand synthesis supermix (Invitrogen, 18080–450) with an oligo dT primer, which was used as template for high-throughput sequencing.

### ddPCR

Genomic DNA was purified from tissue using Beckman gDNAdvance kit (Beckman Coulter, A48705) according to the manufacturer’s instructions and used as template for ddPCR. ddPCR was carried out using ddPCR Supermix for Probes (BioRad, 1863026) with 10 ng of genomic DNA as template and 3 units NEB EcoRI-HF (R3101S) per reaction. Droplets were autogenerated and PCR was performed at an annealing and extension temperature of 61 °C for 2 min for a total of 60 cycles. Droplets were analysed on a QX200 droplet analyser and droplet fluorescence was quantified using QuantaSoft (BioRad). The sequence of primers and probes used are listed in Supplementary Table 4.

### Calculation of targetable genomic adenosines

A custom Python script (Supplementary Code) was used to analyse the targetability of all adenosines in the hg38 human reference genome. An adenosine was counted as targetable if the surrounding genomic sequence context contained a small ABE8e-targetable PAM that would place that adenosine within an appropriate base editing window. The PAM sequences, protospacer lengths and base editing windows associated with each small ABE8e variant are provided in Supplementary Table 5a. The percentage of calculated genomic adenines on each chromosome is shown in Supplementary Table 5b.

### Blood collection and plasma analysis

Initial blood samples were collected following a 4 h fast. Age-matched littermates/colonymates were randomly assigned to experimental groups and administered AAV particles (*n* = 5) via retro-orbital injection. Blood samples were collected following a 4 h fast at 1-week intervals via the tail tip. After 4 weeks, all mice were killed by carbon dioxide asphyxiation after a 4 h fast. Whole livers were collected for genomic DNA isolation and analysis and for hematoxylin/eosin staining, and terminal blood samples were collected.

Pre-treatment and post-treatment plasma human PCSK9, mouse Pcsk9 or mouse ANGPTL3 were measured using the human proprotein convertase 9/PCSK9 Quantikine ELISA kit, mouse proprotein convertase 9/PCSK9 Quantikine ELISA kit or human angiopoietin-like 3 Quantikine ELISA kit, respectively, according to the manufacturer’s instructions (R&D Systems). Total cholesterol or triglyceride levels were measured using the Infinity cholesterol reagent or Infinity triglycerides reagent, respectively, according to the manufacturer’s instructions (Thermo Fisher).

### Liver tissue fixation and histology

A portion of the left medial lobe was fixed in 4% paraformaldehyde at 4 °C overnight, washed with PBS, then dehydrated gradually by serial substitution of PBS for 30%, 50%, 70% and 100% ethanol. Samples were kept at −20 °C until analysis, when they were paraffinized by the Rodent Histopathology Core of Harvard Medical School. Liver paraffin block was then cut into 5 μm sections followed by hematoxylin and eosin staining for histopathological examination.

### Alkaline gel electrophoresis of AAV genomes

Alkaline agarose gel (1% agarose in water with 50 mM NaOH and 1 mM EDTA) was prepared by dissolving agarose in water, allowing to cool but not solidify, then adding a 50× solution of NaOH and EDTA. The formed gel was submerged in 1× alkaline running buffer (50 mM NaOH, 1 mM EDTA) in a submarine style gel electrophoresis setup at 4 °C. AAV (5 × 10^10^ vg) was treated with DNAse I (NEB, M0303S), lysed in 1× alkaline lysis buffer (50 mM NaOH, 1 mM EDTA, 0.3% SDS, 5% glycerol and 0.0025% xylene cyanol) for 3 min at 95 °C, then cooled on ice. Samples were loaded onto the gel, then electrophoresed at 20 V for 15 h. The gel was neutralized in 0.1 M Tris at pH 8 for 1 h at 4 °C with rocking. The gel was stained in 4× SYBR Gold in 0.1 M NaCl at 4 °C with rocking and protection from light. The gel was briefly washed with deionized water, then imaged on a UV transilluminator.

### Statistical analysis

Data are presented as mean ± s.e.m. unless otherwise noted. The number of independent replicates and statistical tests are described in the figure legends. All statistical tests were conducted using GraphPad Prism 9.

### Reporting summary

Further information on research design is available in the Nature Research Reporting Summary linked to this article.

## Supplementary information

Supplementary Information Supplementary figures, tables, code and references.

Reporting Summary

## Data Availability

The data supporting the results in this study are available within the paper and its Supplementary Information. All unmodified reads for sequencing-based data are available from the NCBI Sequence Read Archive, under accession number PRJNA798016. AAV genome sequences are provided in the Supplementary Information. Key plasmids from this work will be available from Addgene (depositor: D.R.L.), and other plasmids and raw data are available from the corresponding author on request.

## Code availability

The custom code for the quantification of targetable genomic adenines is available in the Supplementary Information.
