# Publisher Correction: Efficient in vivo base editing via single adenoassociated viruses with size-optimized genomes encoding compact adenine base editors

**DOI:** 10.1038/s41551-022-00934-x

**Published:** 2022-08-05

**Authors:** Jessie R. Davis, Xiao Wang, Isaac P. Witte, Tony P. Huang, Jonathan M. Levy, Aditya Raguram, Samagya Banskota, Nabil G. Seidah, Kiran Musunuru, David R. Liu

**Affiliations:** 1 Merkin Institute of Transformative Technologies in Healthcare, Broad Institute of MIT and Harvard, Cambridge, MA USA; 2 Department of Chemistry and Chemical Biology, Harvard University, Cambridge, MA USA; 3 Howard Hughes Medical Institute, Harvard University, Cambridge, MA USA; 4 Cardiovascular Institute, Perelman School of Medicine at the University of Pennsylvania, Philadelphia, PA USA; 5 Division of Cardiovascular Medicine, Department of Medicine, Perelman School of Medicine at the University of Pennsylvania, Philadelphia, PA USA; 6 Department of Genetics, Perelman School of Medicine at the University of Pennsylvania, Philadelphia, PA USA; 7 Laboratory of Biochemical Neuroendocrinology, Montreal Clinical Research Institute (IRCM), University of Montreal, Montreal, Quebec Canada

**Keywords:** Genetic engineering, Gene delivery

Correction to: *Nature Biomedical Engineering* 10.1038/s41551-022-00911-4, published online 28 July 2022.

In the version of this article initially published, individual data points in Fig. 5f–k were omitted, and have now been restored in the HTML and PDF versions of the paper.

