# Supplementary material for: Efficient in vivo base editing via single adeno-associated viruses with size-optimized genomes encoding compact adenine base editors
Source: Nat Biomed Eng. 2022 Jul 28;6(11):1272–83. doi: 10.1038/s41551-022-00911-4 (PMC9652153; doi:10.1038/s41551-022-00911-4)
Supplement: Supplementary file 1 — Supplementary figures, tables, code and references. [file 41551_2022_911_MOESM1_ESM.pdf]

nature portfolio

https://doi.org/10.1038/s41551-022-00911-4

Supplementary information

Efficient in vivo base editing via single adeno-associated viruses with size-optimized genomes encoding compact adenine base editors

In the format provided by the authors and unedited

Contents

Supplementary Fig. 1 l Validation of SaABE and SaKKH-ABE targets in mouse Neuro-2A and 3T3 cells

Supplementary Fig. 2 l Titration of sgRNA *in vivo*

Supplementary Fig. 3 l Editing window and indels of SaABE8e *in vivo*.

Supplementary Fig. 4 l Validating guide RNAs targeting *PCSK9* in HEK293T cells and *Pcsk9* and *Angptl3* in Neuro-2A cells

Supplementary Fig. 5 l Alkaline gel electrophoresis of packaged AAV genomes

Supplementary Fig. 6 l *In vivo* editing of control AAVs for lipid modification experiments

Supplementary Fig. 7 l Dose response of single-AAV8 SaKKH-ABE8e and dual-AAV8 SpABE8e on plasma Pcsk9 and total cholesterol

Supplementary Fig. 8 l Raw (unnormalized) levels of plasma analytes of either single-AAV ABE or non-targeting control dual-AAV ABE mice for human PCSK9 and mouse Angptl3 targets

Supplementary Fig. 9 l Histopathological assessment by hematoxylin and eosin staining of livers from mice treated with single-AAV ABE

Supplementary sequences l Sequences of single AAVs used in this study

Supplementary Table 1 l sgRNA protospacer and PAM sequences used in this manuscript

Supplementary Table 2 l sgRNA scaffolds used in this manuscript.

Supplementary Table 3 l Primer sequences used to amplify genomic DNA and cDNA for high throughput sequencing.

Supplementary Table 4 l Primer and probe sequences used for ddPCR

Supplementary Table 5 l Summary of the base editing activity windows of size-minimized ABEs developed in this manuscript and the percentages of targetable genomic adenines.

Supplementary code l Custom python script for calculating the percent of targetable adenines with small-Cas ABEs in the human genome.

Supplementary references

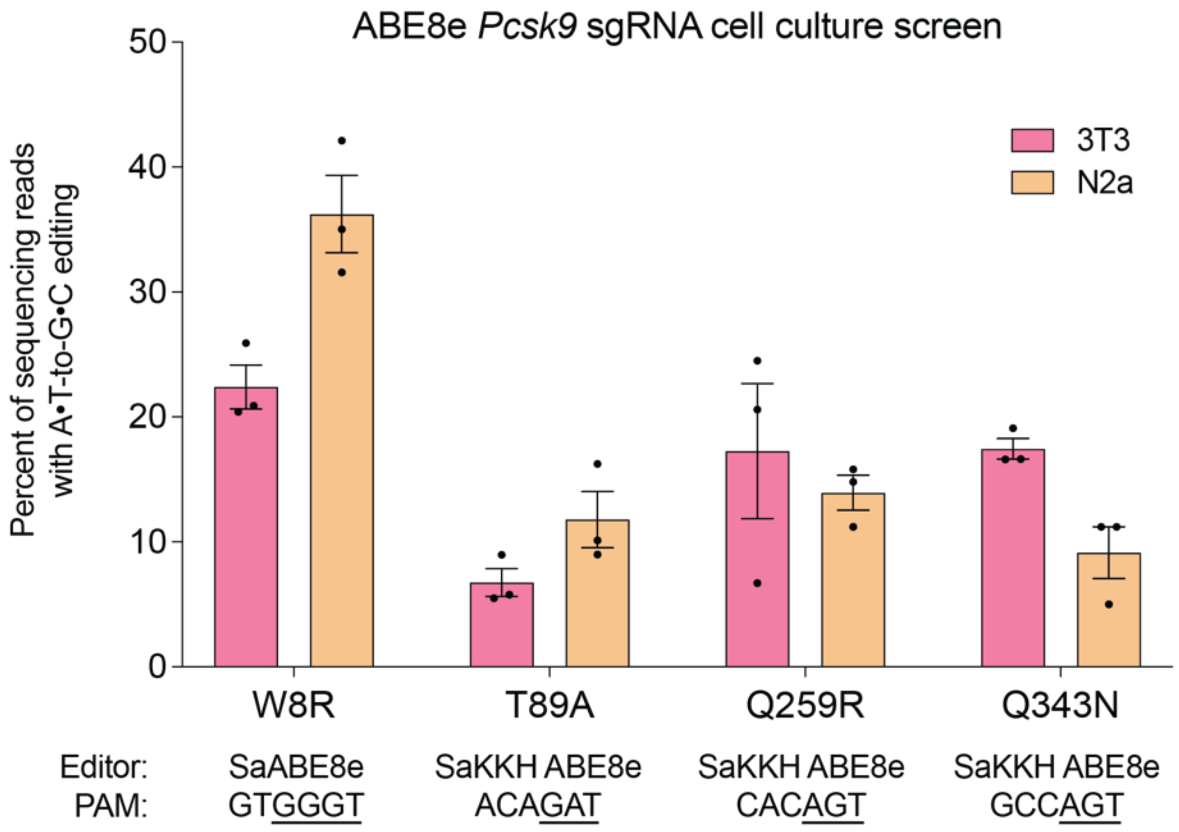

**Supplementary Fig. 1 | ** Validation of SaABE targets in mouse Neuro-2A and 3T3 cells. The base editor and PAM are noted below each set of bars. Dots represent independent biological replicates (n=3) and error bars show SEM.

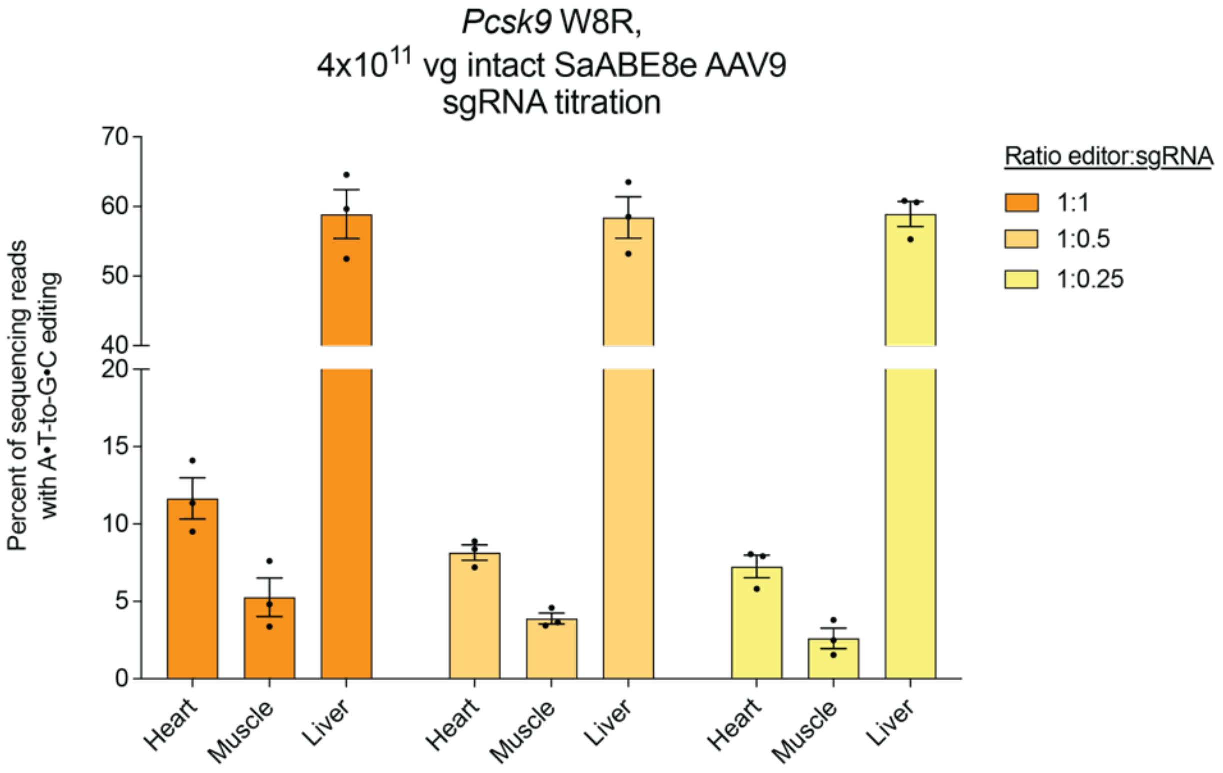

Supplementary Fig. 2 l Titration of sgRNA cassette AAV *in vivo*. A constant dose of $4x10^{11}$ vg of full-length SaABE8e editor AAV was delivered with varying ratios of sgRNA AAV by retroorbital injection to C57BL/6 mice. Tissues were harvested three weeks post injection and analyzed by HTS. Dots represent individual mice (n=3) and error bars show SEM.

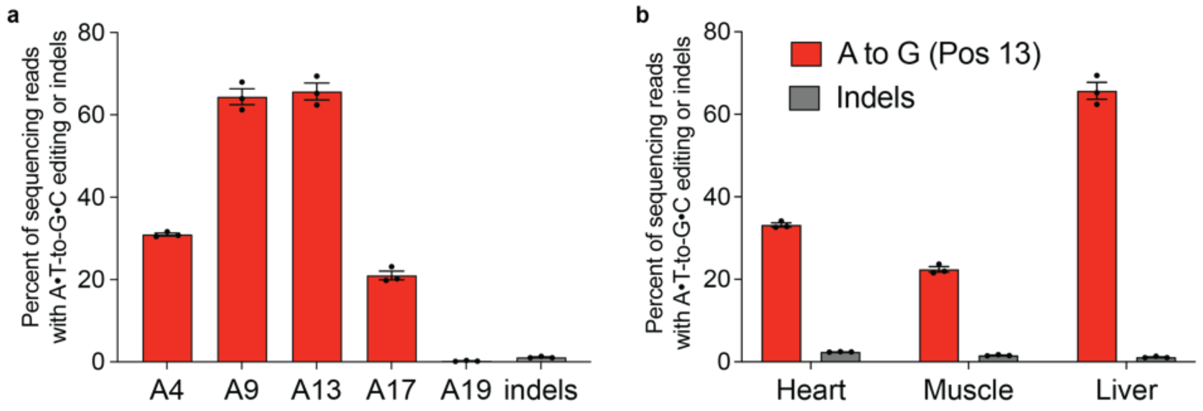

Supplementary Fig. 3 | a, SaABE8e activity window at *Pcsk9* W8R in liver. SaABE8e maintains a wide editing window *in vivo*, consistent with observations in cultured cells. **b**, Indels remain low under all conditions, reaching 2.4%, 1.6%, and 1.1% indels in heart, muscle, and liver, respectively at a high dose of $8x10^{11}$ vg single AAV SaABE8e. Dots represent individual mice (n=3) and error bars show SEM.

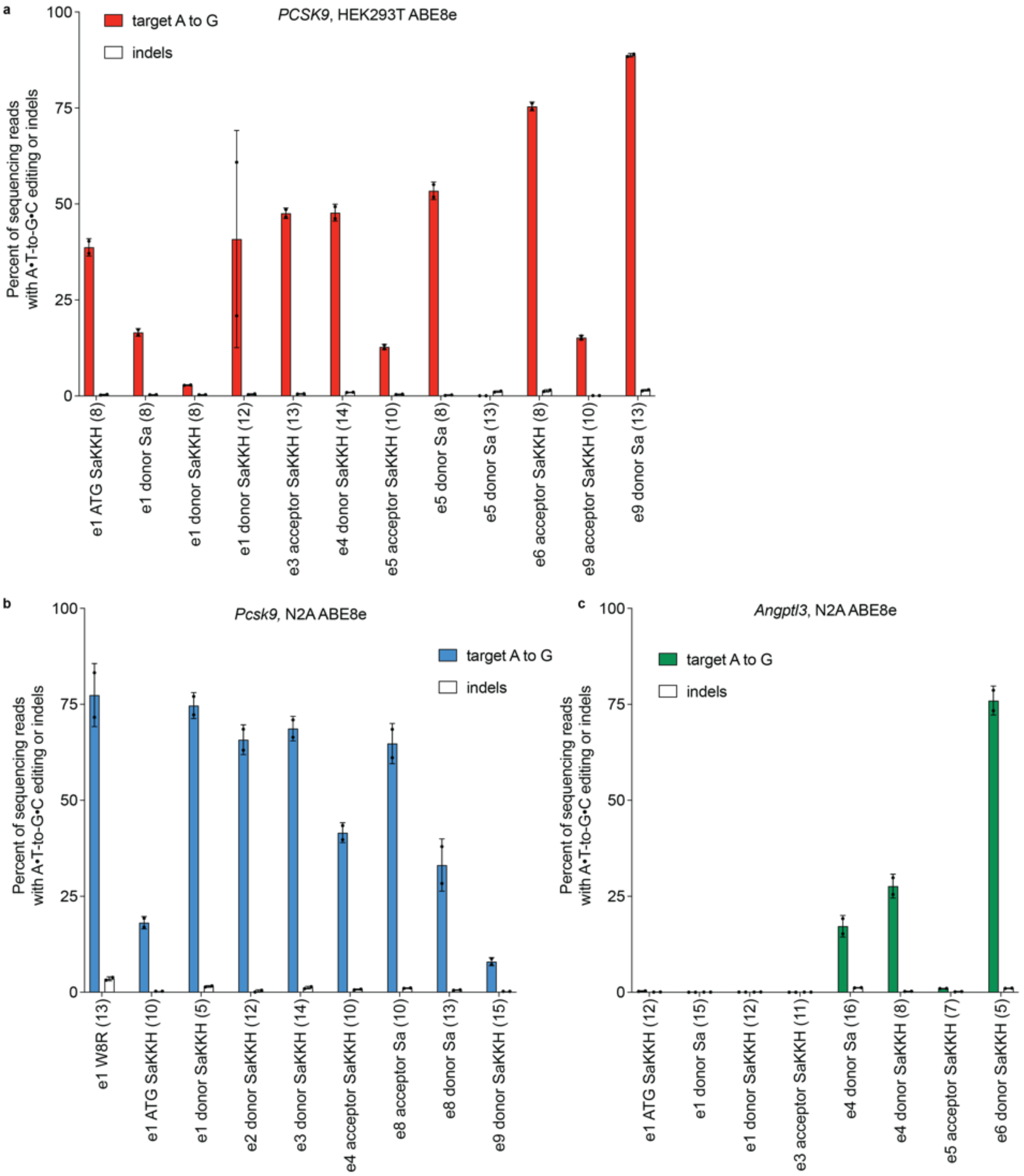

Supplementary Fig. 4 | ** Validating guide RNAs targeting **a, *PCSK9* in HEK293T cells and **b**, *Pcsk9* and **c**, *Angptl3* in mouse Neuro-2a cells. For each sgRNA, the exon, target type (start codon, splice donor, or splice acceptor), ABE8e variant (SaABE8e or SaKKH-ABE8e), and protospacer position that disrupts the indicated target with respect to a 22nt protospacer length are indicated. Editing at the protospacer position that disrupts the indicated target is plotted. Dots represent independent replicates (n=2) and error bars show SD.

Lane	Sample	Expected length (bp)
1	1kb+ ladder	
2	AAV8/SaKKH-ABE8e NpuN	3,415
3	AAV8/SaKKH-ABE8e NpuC U6 Pcsk9 e1 donor	2,976
4	AAV8/SaKKH-ABE8e U6 Pcsk9 e1 donor	5,064
5	AAV8/Sauri ABE8e U6 Pcsk9 e1 donor	5,088

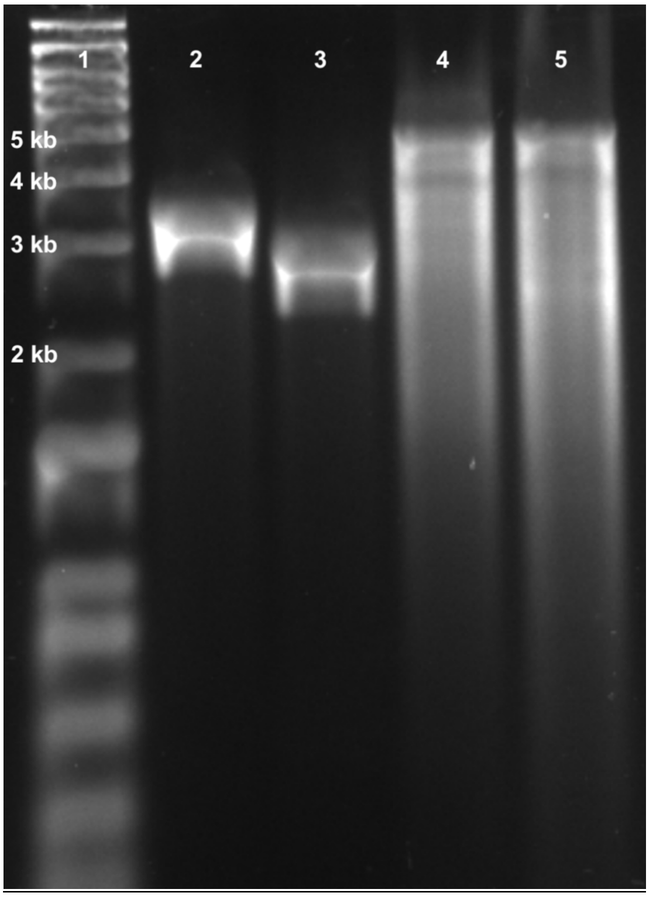

Supplementary Fig. 5 l Alkaline gel electrophoresis of packaged AAV genomes.

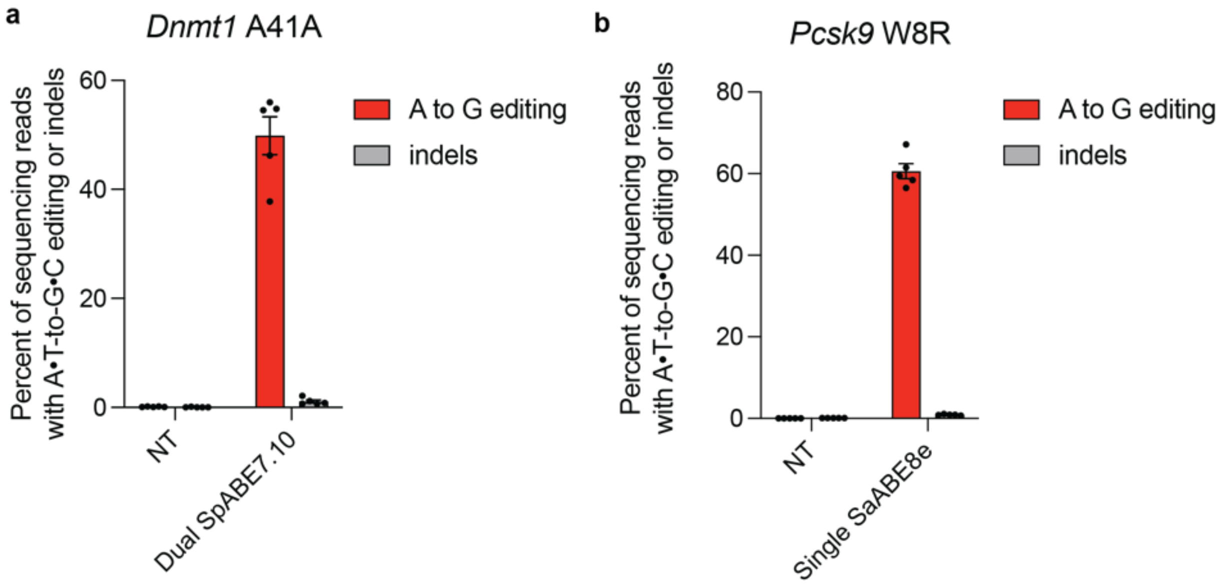

Supplementary Fig. 6 | *In vivo* editing of control AAVs for lipid modification experiments. 6- to 8-week-old C57BL/6 mice were injected by retroorbital injection and whole liver was analyzed by HTS after four weeks. **a**, Editing of *Dnmt1* A41A (silent edit) with dual SpABE7.10 at a dose of $1x10^{11}$ vg dual AAV8. **b**, Installation of *Pcsk9* W8R using single SaKKH-ABE8e at a dose of $1x10^{11}$ vg single AAV8.

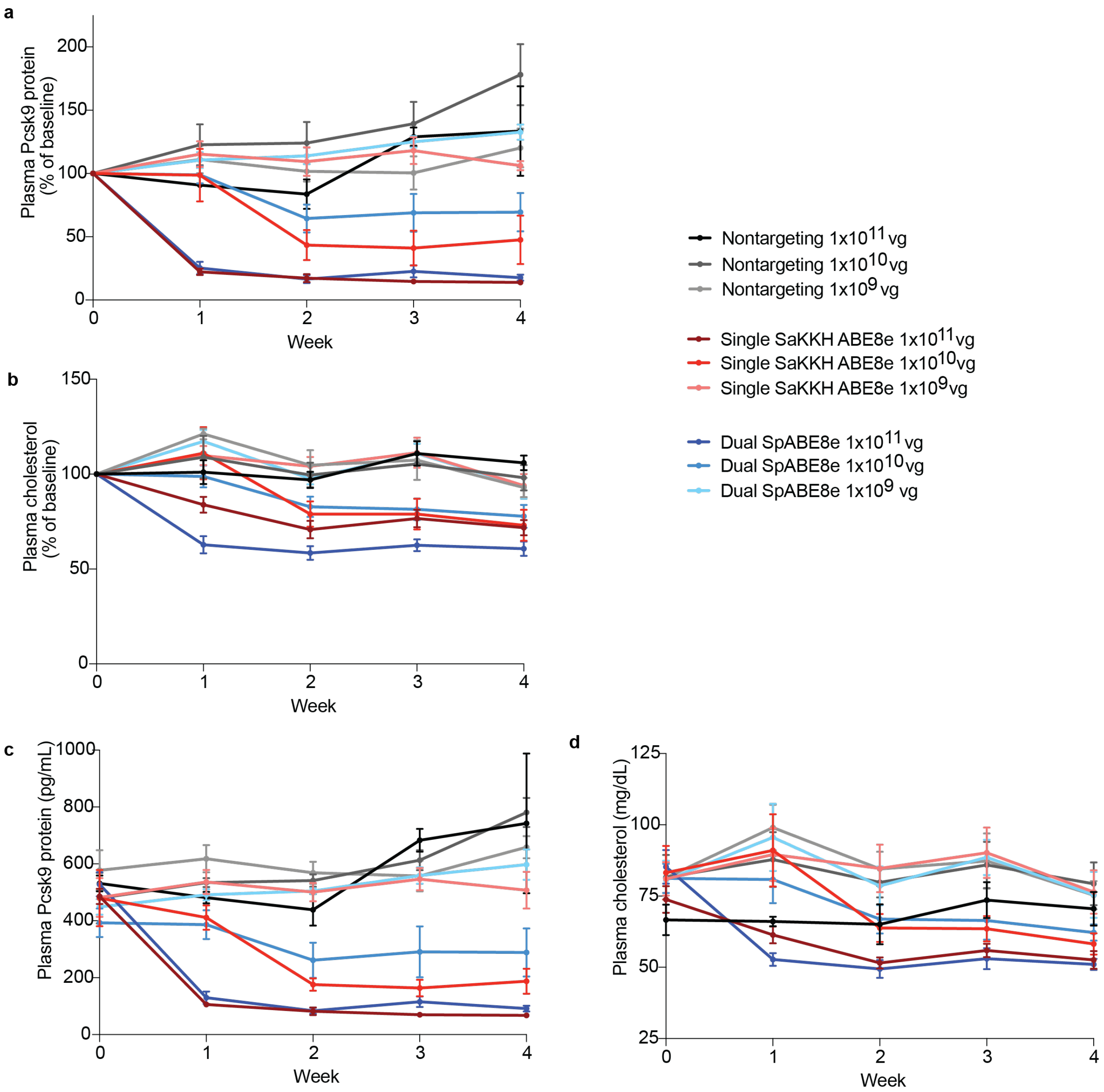

Supplementary Fig. 7 l Dose response of single-AAV8 SaKKH-ABE8e and dual-AAV8 SpABE8e on plasma Pcsk9 and total cholesterol. **a**, Circulating Pcsk9 protein and **b**, total cholesterol from plasma taken weekly, normalized to baseline. **c**, Circulating Pcsk9 protein and **d**, total cholesterol from plasma taken weekly, raw (unnormalized). Dots represent mean values and error bars represent SEM of n=5 different mice. All mice were administered the total dose of AAV8 indicated in the legend systemically by retro-orbital injection at 6-8 weeks of age and blood samples were removed serially over four weeks.

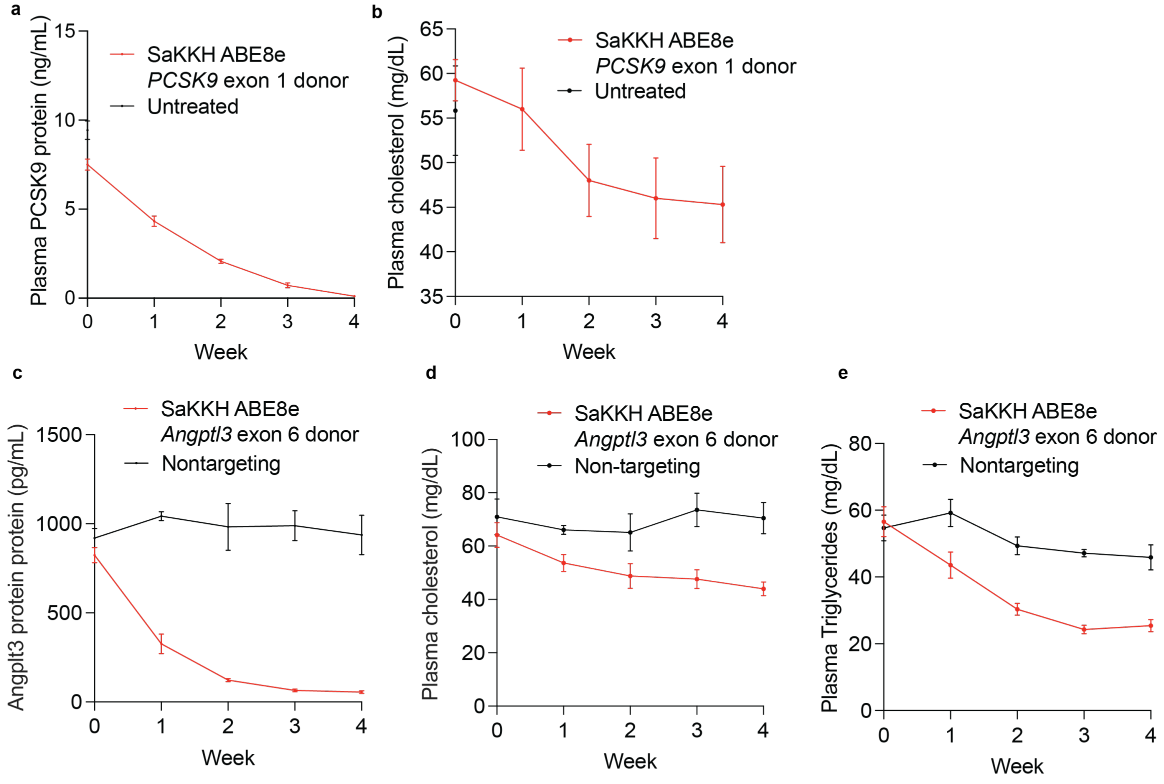

Supplementary Fig. 8 l Raw (unnormalized) levels of plasma analytes of either single-AAV ABE or non-targeting control dual-AAV ABE mice for human PCSK9 and mouse Angptl3 targets. **a**, ELISA of human PCSK9 in plasma from humanized mice. **b**, Total plasma cholesterol in humanized PCSK9 mice. **c**, ELISA of mouse Angptl3 in plasma from C57BL/6 mice. **d**, Total plasma cholesterol in C57BL/6 mice. **e**, Plasma triglycerides from C57BL/6 mice. Dots represent mean values and error bars represent SEM of n=5 different mice. Non-targeting control is dual-AAV ABE7.10 targeting *Dnmt1*. All mice were administered a dose of 1×10^{11} vg AAV8 systemically by retro-orbital injection at 6-8 weeks of age and blood samples were removed serially over four weeks.

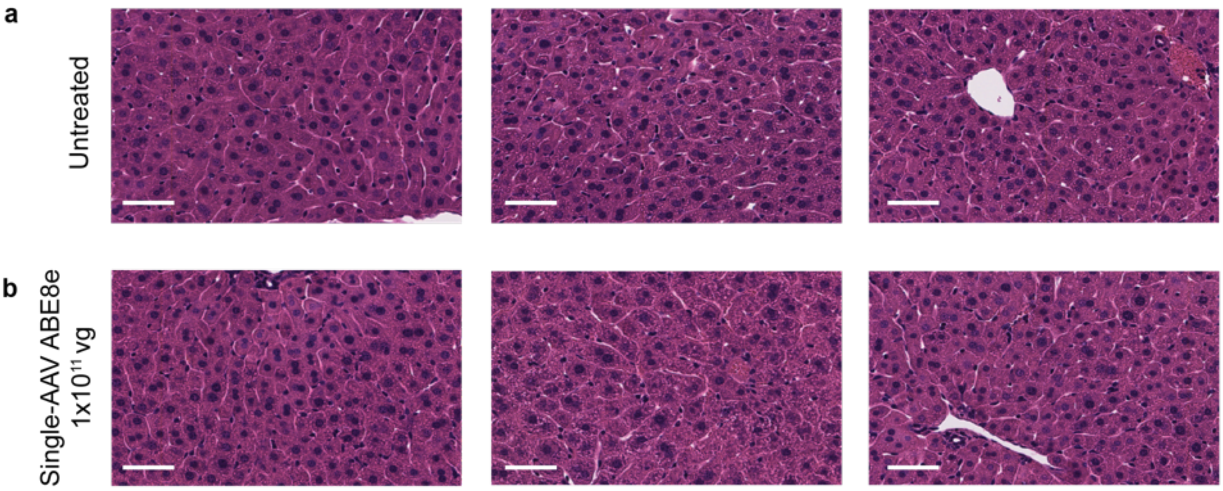

Supplementary Fig. 9 l Histopathological assessment by hematoxylin and eosin staining of livers from **a**, untreated mice and **b**, mice four weeks after treatment with $1x10^{11}$ vg of single AAV8 SaKKH-ABE8e targeting human *PCSK9*. Representative images are shown. Scale bar, 50 μm.

Supplementary sequences l Sequences of single AAVs used in this study.

Sequence of single AAV SaABE8e (5' to 3'), 5,064 bp

ITR-EFS promoter-SaABE8e (start codon-BPNLS-TadA-SaCas9 D10A-BPNLS-stop codon)-bGH polyA-sgRNA (protospacer in **bold**)-U6-ITR (sequences between in grey contain restriction sites for cloning)

CTGCGCGCTCGCTCGCTCACTGAGGCCGCCCGGGCAAAGCCCGGGCGTCGGGCGACCTTTGGTCGC
CCGGCCTCAGTGAGCGAGCGAGCGCGCAGAGAGGGAGTGGCCAACTCCATCACTAGGGGTTCCTGCG
GCCTCTAGAATTCGCTAGCTAGGTCTTGAAAGGAGTGGGAATTGGCTCCGGTGCCCGTCAGTGGGCAG
AGCGCACATCGCCCACAGTCCCCGAGAAGTTGGGGGGAGGGGTCGGCAATTGATCCGGTGCCTAGAG
AAGGTGGCGCGGGGTAAACTGGGAAAGTGATGTCGTGTACTGGCTCCGCCTTTTTCCCGAGGGTGGG
GGAGAACCGTATATAAGTGCAGTAGTCGCCGTGAACGTTCTTTTTCGCAACGGGTTTGCCGCCAGAACA
CAGGACCGGTGCCACCATGAAACGGACAGCCGACGGAAGCGAGTTCGAGTCACCAAAGAAGAAGCGG
AAAGTCTCTGAGGTGGAGTTTTCCCACGAGTACTGGATGAGACATGCCCTGACCCTGGCCAAGAGGGC
ACGGGATGAGAGGGAGGTGCCTGTGGGAGCCGTGCTGGTGCTGAACAATAGAGTGATCGGCGAGGGC
TGGAACAGAGCCATCGGCCTGCACGACCCAACAGCCCATGCCGAAATTATGGCCCTGAGACAGGGCG
GCCTGGTCATGCAGAACTACAGACTGATTGACGCCACCCTGTACGTGACATTCGAGCCTTGCGTGATGT
GCGCCGGCGCCATGATCCACTCTAGGATCGGCCGCGTGGTGTTTGGCGTGAGGAACTCAAAAAGAGG
CGCCGCAGGCTCCCTGATGAACGTGCTGAACTACCCCGGCATGAATCACCGCGTCGAAATTACCGAGG
GAATCCTGGCAGATGAATGTGCCGCCCTGCTGTGCGATTTCTATCGGATGCCTAGACAGGTGTTCAATG
CTCAGAAGAAGGCCCAGAGCTCCATCAACTCCGGAGGATCTAGCGGAGGCTCCTCTGGCTCTGAGACA
CCTGGCACAAGCGAGAGCGCAACACCTGAAAGCAGCGGGGGCAGCAGCGGGGGGTCAGGGAAGCGA
AATTACATTCTGGGGCTGGCCATTGGCATTACATCAGTGGGCTATGGCATCATTGACTACGAGACAAGG
GACGTGATCGACGCCGGCGTGAGACTGTTCAAGGAGGCCAACGTGGAGAACAATGAGGGCCGGAGAT
CCAAGAGGGGAGCAAGGCGCCTGAAGCGGAGAAGGCGCCACAGAATCCAGAGAGTGAAGAAGCTGCT
GTTCGATTACAACCTGCTGACCGACCACTCCGAGCTGTCTGGCATCAATCCTTATGAGGCCAGAGTGAA
GGGCCTGTCCCAGAAGCTGTCTGAGGAGGAGTTTAGCGCCGCCCTGCTGCACCTGGCAAAGAGGAGA
GGCGTGCACAACGTGAATGAGGTGGAGGAGGACACCGGCAACGAGCTGTCCACAAAGGAGCAGATCA
GCCGCAATTCCAAGGCCCTGGAGGAGAAGTATGTGGCCGAGCTGCAGCTGGAGCGGCTGAAGAAGGA
TGGCGAGGTGAGGGGCTCCATCAATCGCTTCAAGACCTCTGACTACGTGAAGGAGGCCAAGCAGCTGC
TGAAGGTGCAGAAGGCCTACCACCAGCTGGATCAGTCCTTTATCGATACATATATCGACCTGCTGGAGA
CAAGGCGCACATACTATGAGGGACCAGGAGAGGGCTCTCCCTTCGGCTGGAAGGACATCAAGGAGTG
GTACGAGATGCTGATGGGCCACTGCACCTATTTTCCAGAGGAGCTGAGAAGCGTGAAGTACGCCTATA
ACGCCGATCTGTACAACGCCCTGAATGACCTGAACAACCTGGTCATCACCAGGGATGAGAACGAGAAG
CTGGAGTACTATGAGAAGTTCCAGATCATCGAGAACGTGTTCAAGCAGAAGAAGAAGCCTACACTGAAG
CAGATCGCCAAGGAGATCCTGGTGAACGAGGAGGACATCAAGGGCTACCGCGTGACCTCCACAGGCA
AGCCAGAGTTCACCAATCTGAAGGTGTATCACGATATCAAGGACATCACAGCCCGGAAGGAGATCATC
GAGAACGCCGAGCTGCTGGATCAGATCGCCAAGATCCTGACCATCTATCAGAGCTCCGAGGACATCCA
GGAGGAGCTGACCAACCTGAATAGCGAGCTGACACAGGAGGAGATCGAGCAGATCAGCAATCTGAAG
GGCTACACCGGCACACACAACCTGAGCCTGAAGGCCATCAATCTGATCCTGGATGAGCTGTGGCACAC
AAACGACAATCAGATCGCCATCTTTAACCGGCTGAAGCTGGTGCCAAAGAAGGTGGACCTGTCCCAGC
AGAAGGAGATCCCAACCACACTGGTGGACGATTTCATCCTGTCTCCCGTGGTGAAGCGGAGCTTCATC
CAGAGCATCAAAGTGATCAACGCCATCATCAAGAAGTACGGCCTGCCCAATGATATCATCATCGAGCTG
GCCAGGGAGAAGAACTCCAAGGACGCCCAGAAGATGATCAATGAGATGCAGAAGAGGAACCGCCAGA
CCAATGAGCGGATCGAGGAGATCATCAGAACCACAGGCAAGGAGAACGCCAAGTACCTGATCGAGAAG
ATCAAGCTGCACGATATGCAGGAGGGCAAGTGTCTGTATTCTCTGGAGGCCATCCCTCTGGAGGACCT
GCTGAACAATCCATTCAACTACGAGGTGGATCACATCATCCCCCGGAGCGTGAGCTTCGACAATTCTTT
TAACAATAAGGTGCTGGTGAAGCAGGAGGAGAACAGCAAGAAGGGCAATAGGACCCCTTTCCAGTACC
TGTCTAGCTCCGATTCTAAGATCAGCTACGAGACATTCAAGAAGCACATCCTGAATCTGGCCAAGGGCA
AGGGCCGCATCAGCAAGACCAAGAAGGAGTACCTGCTGGAGGAGCGGGACATCAACAGATTCTCCGT
GCAGAAGGACTTCATCAACCGGAATCTGGTGGACACCAGATACGCCACACGCGGCCTGATGAATCTGC
TGCGGTCTTATTTCAGAGTGAACAATCTGGATGTGAAGGTGAAGAGCATCAACGGCGGCTTCACCTCCT
TTCTGCGGAGAAAGTGGAAGTTTAAGAAGGAGCGCAACAAGGGCTATAAGCACCACGCCGAGGATGCC
CTGATCATCGCCAATGCCGACTTCATCTTTAAGGAGTGGAAGAAGCTGGACAAGGCCAAGAAAGTGATG
GAGAACCAGATGTTCGAGGAGAAGCAGGCCGAGAGCATGCCCGAGATCGAGACAGAGCAGGAGTACA
AGGAGATTTTCATCACACCTCACCAGATCAAGCACATCAAGGACTTCAAGGACTACAAGTATTCTCACAG
GGTGGATAAGAAGCCCAACCGCGAGCTGATCAATGACACCCTGTATAGCACACGGAAGGACGATAAGG
GCAATACCCTGATCGTGAACAATCTGAACGGCCTGTACGACAAGGATAATGACAAGCTGAAGAAGCTGA
TCAACAAGTCTCCCGAGAAGCTGCTGATGTACCACCACGATCCTCAGACATATCAGAAGCTGAAGCTGA

TCATGGAGCAGTACGGCGACGAGAAGAACCCACTGTATAAGTACTATGAGGAGACAGGCAACTACCTG
ACAAAGTATAGCAAGAAGGATAATGGCCCCGTGATCAAGAAGATCAAGTACTATGGCAACAAGCTGAAT
GCCCACCTGGACATCACCGACGATTACCCTAACTCTCGCAATAAGGTGGTGAAGCTGAGCCTGAAGCC
ATACCGGTTCGACGTGTACCTGGACAACGGCGTGTATAAGTTTGTGACAGTGAAGAATCTGGATGTGAT
CAAGAAGGAGAACTACTATGAGGTGAACAGCAAGTGCTACGAGGAGGCCAAGAAGCTGAAGAAGATCA
GCAACCAGGCCGAGTTCATCGCCTCTTTTTACAACAATGACCTGATCAAGATCAATGGCGAGCTGTATA
GAGTGATCGGCGTGAACAATGATCTGCTGAACAGAATCGAAGTGAATATGATCGACATCACCTACAGGG
AGTATCTGGAGAACATGAATGATAAGAGGCCCCCTCGCATCATCAAGACCATCGCCTCTAAGACACAGA
GCATCAAGAAGTACAGCACAGACATCCTGGGGAACCTGTATGAAGTCAAGAGCAAGAAACATCCTCAGA
TTATCAAGAAAGGCTCTGGCGGCTCAAAAAGAACCGCCGACGGCAGCGAATTCGAGCCCAAGAAGAAG
AGGAAAGTCTAATAGATCTCGACTGTGCCTTCTAGTTGCCAGCCATCTGTTGTTTGCCCCTCCCCCGTG
CCTTCCTTGACCCTGGAAGGTGCCACTCCCACTGTCCTTTCCTAATAAAATGAGGAAATTGCATCGCATT
GTCTGAGTAGGTGTCATTCTATTCTGGGGGGTGGGGTGGGGCAGGACAGCAAGGGGGAGGATTGGGA
AGACAATAGCAGGCATGCTGGGGATGCGGTGGGCTCTATGGCTCGAGCGGCCCAAGCTTAAAAAAATC
TCGCCAACAAGTTGACGAGATAAACACGGCATTTTGCCTTGTTTTAGTAGATTCTGTAATTTTCATTACAG
AGTACTAAAAC**CGCCGTTGCTCCAAGGTATGGC**GGTGTTTCGTCCTTTCCACAAGATATATAAAGCCAA
GAAATCGAAATACTTTCAAGTTACGGTAAGCATATGATAGTCCATTTTAAAACATAATTTTAAAACTGCAA
ACTACCCAAGAAATTATTACTTTCTACGTCACGTATTTTGTACTAATATCTTTGTGTTTACAGTCAAATTAA
TTCTAATTATCTCTCTAACAGCCTTGTATCGTATATGCAAATATGAAGGAATCATGGGAAATAGGCCCTC
TTCCTGCCCGACCTTGCGGCCGCCTGCGCGCTCGCTCGCTCACTGAGGCCGCCCGGGCAAAGCCCGG
GCGTCGGGCGACCTTTGGTCGCCCGGCCTCAGTGAGCGAGCGAGCGCGCAGAGAGGGAGTGGCCAA
CTCCATCACTAGGGGTTCCT

Sequence of single AAV SaKKHABE8e (5’ to 3’), 5,064 bp

ITR-EFS promoter-SaKKHABE8e (start codon-BPNLS-TadA-SaKKHCas9 D10A-BPNLS-stop codon)-bGH polyA-sgRNA (protospacer in **bold**)-U6-ITR (sequences between in grey contain restriction sites for cloning)

CTGCGCGCTCGCTCGCTCACTGAGGCCGCCCGGGCAAAGCCCGGGCGTCGGGCGACCTTTGGTCGC
CCGGCCTCAGTGAGCGAGCGAGCGCGCAGAGAGGGAGTGGCCAACTCCATCACTAGGGGTTCCTGCG
GCCTCTAGAATTCGCTAGCTAGGTCTTGAAAGGAGTGGGAATTGGCTCCGGTGCCCGTCAGTGGGCAG
AGCGCACATCGCCCACAGTCCCCGAGAAGTTGGGGGGAGGGGTCGGCAATTGATCCGGTGCCTAGAG
AAGGTGGCGCGGGGTAAACTGGGAAAGTGATGTCGTGTACTGGCTCCGCCTTTTTCCCGAGGGTGGG
GGAGAACCGTATATAAGTGCAGTAGTCGCCGTGAACGTTCTTTTTCGCAACGGGTTTGCCGCCAGAACA
CAGGACCGGTGCCACCATGAAACGGACAGCCGACGGAAGCGAGTTCGAGTCACCAAAGAAGAAGCGG
AAAGTCTCTGAGGTGGAGTTTTCCCACGAGTACTGGATGAGACATGCCCTGACCCTGGCCAAGAGGGC
ACGGGATGAGAGGGAGGTGCCTGTGGGAGCCGTGCTGGTGCTGAACAATAGAGTGATCGGCGAGGGC
TGGAACAGAGCCATCGGCCTGCACGACCCAACAGCCCATGCCGAAATTATGGCCCTGAGACAGGGCG
GCCTGGTCATGCAGAACTACAGACTGATTGACGCCACCCTGTACGTGACATTCGAGCCTTGCGTGATGT
GCGCCGGCGCCATGATCCACTCTAGGATCGGCCGCGTGGTGTTTGGCGTGAGGAACTCAAAAAGAGG
CGCCGCAGGCTCCCTGATGAACGTGCTGAACTACCCCGGCATGAATCACCGCGTCGAAATTACCGAGG
GAATCCTGGCAGATGAATGTGCCGCCCTGCTGTGCGATTTCTATCGGATGCCTAGACAGGTGTTCAATG
CTCAGAAGAAGGCCCAGAGCTCCATCAACTCCGGAGGATCTAGCGGAGGCTCCTCTGGCTCTGAGACA
CCTGGCACAAGCGAGAGCGCAACACCTGAAAGCAGCGGGGGCAGCAGCGGGGGGTCAGGGAAGCGA
AATTACATTCTGGGGCTGGCCATTGGCATTACATCAGTGGGCTATGGCATCATTGACTACGAGACAAGG
GACGTGATCGACGCCGGCGTGAGACTGTTCAAGGAGGCCAACGTGGAGAACAATGAGGGCCGGAGAT
CCAAGAGGGGAGCAAGGCGCCTGAAGCGGAGAAGGCGCCACAGAATCCAGAGAGTGAAGAAGCTGCT
GTTCGATTACAACCTGCTGACCGACCACTCCGAGCTGTCTGGCATCAATCCTTATGAGGCCAGAGTGAA
GGGCCTGTCCCAGAAGCTGTCTGAGGAGGAGTTTAGCGCCGCCCTGCTGCACCTGGCAAAGAGGAGA
GGCGTGCACAACGTGAATGAGGTGGAGGAGGACACCGGCAACGAGCTGTCCACAAAGGAGCAGATCA
GCCGCAATTCCAAGGCCCTGGAGGAGAAGTATGTGGCCGAGCTGCAGCTGGAGCGGCTGAAGAAGGA
TGGCGAGGTGAGGGGCTCCATCAATCGCTTCAAGACCTCTGACTACGTGAAGGAGGCCAAGCAGCTGC
TGAAGGTGCAGAAGGCCTACCACCAGCTGGATCAGTCCTTTATCGATACATATATCGACCTGCTGGAGA
CAAGGCGCACATACTATGAGGGACCAGGAGAGGGCTCTCCCTTCGGCTGGAAGGACATCAAGGAGTG
GTACGAGATGCTGATGGGCCACTGCACCTATTTTCCAGAGGAGCTGAGAAGCGTGAAGTACGCCTATA
ACGCCGATCTGTACAACGCCCTGAATGACCTGAACAACCTGGTCATCACCAGGGATGAGAACGAGAAG
CTGGAGTACTATGAGAAGTTCCAGATCATCGAGAACGTGTTCAAGCAGAAGAAGAAGCCTACACTGAAG
CAGATCGCCAAGGAGATCCTGGTGAACGAGGAGGACATCAAGGGCTACCGCGTGACCTCCACAGGCA
AGCCAGAGTTCACCAATCTGAAGGTGTATCACGATATCAAGGACATCACAGCCCGGAAGGAGATCATC

GAGAACGCCGAGCTGCTGGATCAGATCGCCAAGATCCTGACCATCTATCAGAGCTCCGAGGACATCCA
GGAGGAGCTGACCAACCTGAATAGCGAGCTGACACAGGAGGAGATCGAGCAGATCAGCAATCTGAAG
GGCTACACCGGCACACACAACCTGAGCCTGAAGGCCATCAATCTGATCCTGGATGAGCTGTGGCACAC
AAACGACAATCAGATCGCCATCTTTAACCGGCTGAAGCTGGTGCCAAAGAAGGTGGACCTGTCCCAGC
AGAAGGAGATCCCAACCACACTGGTGGACGATTTCATCCTGTCTCCCGTGGTGAAGCGGAGCTTCATC
CAGAGCATCAAAGTGATCAACGCCATCATCAAGAAGTACGGCCTGCCCAATGATATCATCATCGAGCTG
GCCAGGGAGAAGAACTCCAAGGACGCCCAGAAGATGATCAATGAGATGCAGAAGAGGAACCGCCAGA
CCAATGAGCGGATCGAGGAGATCATCAGAACCACAGGCAAGGAGAACGCCAAGTACCTGATCGAGAAG
ATCAAGCTGCACGATATGCAGGAGGGCAAGTGTCTGTATTCTCTGGAGGCCATCCCTCTGGAGGACCT
GCTGAACAATCCATTCAACTACGAGGTGGATCACATCATCCCCCGGAGCGTGAGCTTCGACAATTCTTT
TAACAATAAGGTGCTGGTGAAGCAGGAGGAGAACAGCAAGAAGGGCAATAGGACCCCTTTCCAGTACC
TGTCTAGCTCCGATTCTAAGATCAGCTACGAGACATTCAAGAAGCACATCCTGAATCTGGCCAAGGGCA
AGGGCCGCATCAGCAAGACCAAGAAGGAGTACCTGCTGGAGGAGCGGGACATCAACAGATTCTCCGT
GCAGAAGGACTTCATCAACCGGAATCTGGTGGACACCAGATACGCCACACGCGGCCTGATGAATCTGC
TGCGGTCTTATTTCAGAGTGAACAATCTGGATGTGAAGGTGAAGAGCATCAACGGCGGCTTCACCTCCT
TTCTGCGGAGAAAGTGGAAGTTTAAGAAGGAGCGCAACAAGGGCTATAAGCACCACGCCGAGGATGCC
CTGATCATCGCCAATGCCGACTTCATCTTTAAGGAGTGGAAGAAGCTGGACAAGGCCAAGAAAGTGATG
GAGAACCAGATGTTCGAGGAGAAGCAGGCCGAGAGCATGCCCGAGATCGAGACAGAGCAGGAGTACA
AGGAGATTTTCATCACACCTCACCAGATCAAGCACATCAAGGACTTCAAGGACTACAAGTATTCTCACAG
GGTGGATAAGAAGCCCAACCGCAAGCTGATCAATGACACCCTGTATAGCACACGGAAGGACGATAAGG
GCAATACCCTGATCGTGAACAATCTGAACGGCCTGTACGACAAGGATAATGACAAGCTGAAGAAGCTGA
TCAACAAGTCTCCCGAGAAGCTGCTGATGTACCACCACGATCCTCAGACATATCAGAAGCTGAAGCTGA
TCATGGAGCAGTACGGCGACGAGAAGAACCCACTGTATAAGTACTATGAGGAGACAGGCAACTACCTG
ACAAAGTATAGCAAGAAGGATAATGGCCCCGTGATCAAGAAGATCAAGTACTATGGCAACAAGCTGAAT
GCCCACCTGGACATCACCGACGATTACCCTAACTCTCGCAATAAGGTGGTGAAGCTGAGCCTGAAGCC
ATACCGGTTCGACGTGTACCTGGACAACGGCGTGTATAAGTTTGTGACAGTGAAGAATCTGGATGTGAT
CAAGAAGGAGAACTACTATGAGGTGAACAGCAAGTGCTACGAGGAGGCCAAGAAGCTGAAGAAGATCA
GCAACCAGGCCGAGTTCATCGCCTCTTTTTACAAGAATGACCTGATCAAGATCAATGGCGAGCTGTATA
GAGTGATCGGCGTGAACAATGATCTGCTGAACAGAATCGAAGTGAATATGATCGACATCACCTACAGGG
AGTATCTGGAGAACATGAATGATAAGAGGCCCCCTCATATCATCAAGACCATCGCCTCTAAGACACAGA
GCATCAAGAAGTACAGCACAGACATCCTGGGGAACCTGTATGAAGTCAAGAGCAAGAAACATCCTCAGA
TTATCAAGAAAGGCTCTGGCGGCTCAAAAAGAACCGCCGACGGCAGCGAATTCGAGCCCAAGAAGAAG
AGGAAAGTCTAATAGATCTCGACTGTGCCTTCTAGTTGCCAGCCATCTGTTGTTTGCCCCTCCCCCGTG
CCTTCCTTGACCCTGGAAGGTGCCACTCCCACTGTCCTTTCCTAATAAAATGAGGAAATTGCATCGCATT
GTCTGAGTAGGTGTCATTCTATTCTGGGGGGTGGGGTGGGGCAGGACAGCAAGGGGGAGGATTGGGA
AGACAATAGCAGGCATGCTGGGGATGCGGTGGGCTCTATGGCTCGAGCGGCCCAAGCTTAAAAAAATC
TCGCCAACAAGTTGACGAGATAAACACGGCATTTTGCCTTGTTTTAGTAGATTCTGTAATTTTCATTACAG
AGTACTAAAAC**CGCCGTTGCTCCAAGGTATGGC**GGTGTTTCGTCCTTTCCACAAGATATATAAAGCCAA
GAAATCGAAATACTTTCAAGTTACGGTAAGCATATGATAGTCCATTTTAAAACATAATTTTAAAACTGCAA
ACTACCCAAGAAATTATTACTTTCTACGTCACGTATTTTGTACTAATATCTTTGTGTTTACAGTCAAATTAA
TTCTAATTATCTCTCTAACAGCCTTGTATCGTATATGCAAATATGAAGGAATCATGGGAAATAGGCCCTC
TTCCTGCCCGACCTTGCGGCCGCCTGCGCGCTCGCTCGCTCACTGAGGCCGCCCGGGCAAAGCCCGG
GCGTCGGGCGACCTTTGGTCGCCCGGCCTCAGTGAGCGAGCGAGCGCGCAGAGAGGGAGTGGCCAA
CTCCATCACTAGGGGTTCCT

Sequence of single AAV SauriABE8e (5' to 3'), 5,087 bp

ITR-EFS promoter-SaKKHABE8e (start codon-BPNLS-TadA-SauriCas9 D10A-BPNLS-stop codon)-bGH polyA-sgRNA (protospacer in **bold**)-U6-ITR (sequences between in grey contain restriction sites for cloning)

CTGCGCGCTCGCTCGCTCACTGAGGCCGCCCGGGCAAAGCCCGGGCGTCGGGCGACCTTTGGTCGC
CCGGCCTCAGTGAGCGAGCGAGCGCGCAGAGAGGGAGTGGCCAACTCCATCACTAGGGGTTCCTGCG
GCCTCTAGAATTCGCTAGCTAGGTCTTGAAAGGAGTGGGAATTGGCTCCGGTGCCCGTCAGTGGGCAG
AGCGCACATCGCCCACAGTCCCCGAGAAGTTGGGGGGAGGGGTCGGCAATTGATCCGGTGCCTAGAG
AAGGTGGCGCGGGGTAAACTGGGAAAGTGATGTCGTGTACTGGCTCCGCCTTTTTCCCGAGGGTGGG
GGAGAACCGTATATAAGTGCAGTAGTCGCCGTGAACGTTCTTTTTCGCAACGGGTTTGCCGCCAGAACA
CAGGACCGGTGCCACCATGAAACGGACAGCCGACGGAAGCGAGTTCGAGTCACCAAAGAAGAAGCGG
AAAGTCTCTGAGGTGGAGTTTTCCCACGAGTACTGGATGAGACATGCCCTGACCCTGGCCAAGAGGGC
ACGGGATGAGAGGGAGGTGCCTGTGGGAGCCGTGCTGGTGCTGAACAATAGAGTGATCGGCGAGGGC
TGGAACAGAGCCATCGGCCTGCACGACCCAACAGCCCATGCCGAAATTATGGCCCTGAGACAGGGCG

GCCTGGTCATGCAGAACTACAGACTGATTGACGCCACCCTGTACGTGACATTCGAGCCTTGCGTGATGT
GCGCCGGCGCCATGATCCACTCTAGGATCGGCCGCGTGGTGTTTGGCGTGAGGAACTCAAAAAGAGG
CGCCGCAGGCTCCCTGATGAACGTGCTGAACTACCCCGGCATGAATCACCGCGTCGAAATTACCGAGG
GAATCCTGGCAGATGAATGTGCCGCCCTGCTGTGCGATTTCTATCGGATGCCTAGACAGGTGTTCAATG
CTCAGAAGAAGGCCCAGAGCTCCATCAACTCCGGAGGATCTAGCGGAGGCTCCTCTGGCTCTGAGACA
CCTGGCACAAGCGAGAGCGCAACACCTGAAAGCAGCGGGGGCAGCAGCGGGGGGTCAATGCAGGAG
AACCAGCAGAAGCAGAACTACATCCTGGGCCTGGCCATCGGAATCACCAGCGTCGGCTACGGACTGAT
CGATAGCAAGACAAGAGAAGTGATCGACGCCGGCGTTAGACTCTTTCCAGAAGCTGATAGCGAGAACA
ACTCCAACCGCAGAAGCAAGCGGGGCGCCAGACGGTTAAAACGGAGAAGAATCCACCGGCTGAACCG
GGTCAAAGACCTGCTCGCTGATTACCAGATGATCGATCTTAACAATGTTCCTAAGAGCACCGACCCCTA
CACCATCAGAGTGAAGGGCCTCCGGGAGCCTCTGACAAAAGAAGAATTCGCCATCGCCCTCCTGCATA
TCGCTAAGAGAAGAGGCCTGCACAACATCAGTGTGTCCATGGGCGACGAAGAGCAGGACAATGAACTG
AGCACCAAGCAGCAGCTGCAAAAGAATGCCCAGCAACTGCAGGACAAGTATGTGTGCGAACTGCAGTT
AGAACGGCTGACCAACATCAACAAGGTCAGAGGCGAGAAGAACAGATTTAAGACAGAGGACTTTGTGA
AAGAAGTGAAACAGCTGTGCGAAACCCAGAGACAGTACCACAACATCGACGACCAATTCATCCAGCAGT
ACATCGACCTGGTGTCTACAAGACGGGAGTACTTCGAGGGCCCCGGCAACGGCTCTCCATACGGCTG
GGACGGCGACCTGCTGAAGTGGTACGAGAAGCTGATGGGCAGATGCACCTATTTCCCCGAAGAACTGA
GGTCCGTGAAGTACGCCTACAGCGCCGACCTCTTCAACGCCCTGAACGACCTGAACAACCTCGTTGTG
ACCAGGGATGACAATCCAAAGCTTGAGTACTACGAGAAGTACCACATTATTGAGAACGTGTTCAAGCAA
AAGAAGAATCCCACACTCAAACAAATCGCCAAAGAGATCGGCGTGCAAGATTACGACATCCGGGGCTAT
AGAATCACAAAGAGCGGCAAACCTCAGTTCACCTCTTTTAAGCTGTATCACGACCTGAAGAACATCTTC
GAGCAGGCCAAATACCTGGAAGATGTGGAAATGCTGGACGAGATCGCCAAGATCCTGACCATCTACCA
GGATGAGATTAGCATCAAGAAAGCCCTGGACCAGCTGCCCGAACTGCTGACAGAGAGCGAGAAATCTC
AGATCGCACAGCTCACCGGCTATACAGGCACCCACAGACTGAGCCTGAAGTGCATCCACATTGTGATC
GACGAGCTGTGGGAGAGCCCCGAGAACCAGATGGAAATCTTTACCAGACTGAATCTGAAACCTAAGAA
GGTGGAAATGAGCGAGATCGACAGCATACCCACCACCCTGGTCGACGAGTTCATCCTCTCACCTGTGG
TGAAGCGGGCCTTCATCCAGAGCATCAAGGTAATCAACGCAGTGATCAATCGGTTCGGCCTGCCAGAG
GACATCATCATCGAGCTGGCCAGAGAAAAGAATAGCAAGGATCGGAGAAAGTTCATTAACAAGCTGCAG
AAACAAAATGAGGCCACAAGAAAGAAAATCGAACAGCTGCTGGCCAAGTACGGCAACACCAATGCCAA
GTACATGATCGAGAAGATCAAGCTGCACGACATGCAGGAGGGCAAGTGCCTGTACAGCCTGGAGGCTA
TTCCTCTGGAAGACCTGCTGAGCAACCCGACACACTACGAAGTTGACCACATTATCCCCAGATCTGTGA
GCTTTGACAACAGCCTGAACAACAAAGTGCTGGTGAAACAAAGCGAAAACAGCAAGAAGGGCAATCGC
ACCCCTTACCAGTACCTGAGCAGCAACGAGTCTAAGATTAGCTACAACCAGTTTAAGCAGCACATCCTG
AACCTGAGCAAGGCCAAGGACAGAATCAGCAAGAAAAAAAGAGATATGCTGCTGGAAGAGAGAGATAT
CAACAAGTTCGAAGTGCAGAAGGAATTCATTAACCGGAACCTGGTGGATACACGGTACGCCACCAGAG
AACTGTCTAACCTGCTGAAGACCTACTTCAGCACCCATGACTACGCCGTGAAGGTGAAGACCATCAACG
GCGGCTTCACTAACCACCTGAGGAAGGTGTGGGATTTCAAGAAGCACAGAAACCACGGCTACAAGCAC
CACGCCGAAGATGCCCTGGTGATCGCCAACGCCGACTTCCTGTTTAAGACACATAAGGCCCTGCGGAG
AACCGATAAGATCCTGGAACAACCTGGCCTGGAAGTGAATGATACAACCGTGAAAGTGGACACCGAGG
AAAAATACCAGGAGCTGTTCGAGACACCTAAGCAAGTGAAGAACATCAAGCAGTTCCGGGACTTCAAGT
ACAGCCACCGAGTGGACAAGAAGCCTAACCGGCAGCTTATCAACGACACACTGTACTCCACCAGAGAG
ATTGATGGCGAAACCTACGTGGTGCAGACCCTTAAGGATCTGTACGCCAAGGACAACGAGAAAGTGAA
GAAGCTGTTCACCGAAAGACCTCAGAAGATCCTGATGTACCAGCACGACCCTAAGACCTTCGAGAAACT
GATGACAATCCTGAACCAGTACGCTGAGGCCAAGAACCCTCTGGCTGCTTATTACGAGGACAAAGGCG
AGTACGTGACCAAGTACGCCAAGAAAGGCAATGGACCTGCCATCCACAAGATCAAGTATATCGATAAGA
AGCTTGGATCTTACCTGGATGTTAGCAACAAGTATCCTGAGACACAGAACAAGCTTGTGAAGCTGTCCC
TGAAGAGCTTTAGATTCGACATCTACAAGTGTGAACAGGGCTACAAGATGGTGTCCATCGGATACCTGG
ACGTGCTGAAGAAAGATAACTACTACTACATCCCTAAGGACAAGTACGAGGCCGAGAAGCAGAAAAAGA
AGATCAAGGAATCTGATCTTTTTGTGGGCAGCTTCTACTACAACGACCTCATCATGTACGAGGATGAACT
GTTCAGAGTGATAGGAGTGAACAGCGACATCAACAATCTGGTTGAGCTAAACATGGTCGACATTACCTA
CAAGGACTTCTGCGAGGTGAACAACGTGACAGGCGAGAAAAGAATCAAAAAGACTATCGGCAAGCGCG
TGGTCCTGATCGAGAAGTACACCACAGATATTCTAGGCAACCTGTACAAGACTCCCCTGCCTAAGAAGC
CCCAGCTTATCTTCAAGCGGGGAGAACTGTCTGGCGGCTCAAAAAGAACCGCCGACGGCAGCGAATTC
GAGCCCAAGAAGAAGAGGAAAGTCTAATAGATCTCGACTGTGCCTTCTAGTTGCCAGCCATCTGTTGTT
TGCCCCTCCCCCGTGCCTTCCTTGACCCTGGAAGGTGCCACTCCCACTGTCCTTTCCTAATAAAATGAG
GAAATTGCATCGCATTGTCTGAGTAGGTGTCATTCTATTCTGGGGGGTGGGGTGGGGCAGGACAGCAA
GGGGGAGGATTGGGAAGACAATAGCAGGCATGCTGGGGATGCGGTGGGCTCTATGGCTCGAGCGGC
CCAAGCTTAAAAAAATCTCGCCAACAAGTTGACGAGATAAACACGGCATTTTGCCTTGTTTTAGTAGATT
CTGTAATTTTCATTACAGAGTACTAAAAC**CGTTGCTCCAAGGTATGGGTGC**GGTGTTTCGTCCTTTCCAC
AAGATATATAAAGCCAAGAAATCGAAATACTTTCAAGTTACGGTAAGCATATGATAGTCCATTTTAAAACA

TAATTTTAAAACTGCAAACTACCCAAGAAATTATTACTTTCTACGTCACGTATTTTGTACTAATATCTTTGT
GTTTACAGTCAAATTAATTCTAATTATCTCTCTAACAGCCTTGTATCGTATATGCAAATATGAAGGAATCA
TGGGAAATAGGCCCTCTTCCTGCCCGACCTTGCGGCCGCCTGCGCGCTCGCTCGCTCACTGAGGCCG
CCCGGGCAAAGCCCGGGCGTCGGGCGACCTTTGGTCGCCCGGCCTCAGTGAGCGAGCGAGCGCGCA
GAGAGGGAGTGGCCAACTCCATCACTAGGGGTTCCT

Supplementary Table 1 I sgRNA sequences used in this manuscript.

Fig.	Site ID	Protospacer sequence	PAM
Fig 1	Sa-W8R	GCCACCGCAGCCACGCAGAGCA	GTGGGT
3a	Site 1 TS90-GAPDH	GCAAGAGCACAAGAGGAAGAGAG	AGACCC
3a	Site 2 TS89-SEC61B	GCCCTCATCTCCAATATGGTATGG	CGGCCC
3a	Site 3 TS88-FANCF	GAGGCAAGAGGGCGGCTTTGGGCG	GGGTCC
3a	Site 4 TS72-LINC01588	GACCAGCCCCTCGAAGGCAAGGCC	AGGACC
3a	Site 5 TS81-LSP1	TATGTTCCAGCTTCCTGGGTCTGC	AGGTCC
3a	Site 6 Nme10-EMX1	GGACCCTCTCCATTTCTACCCCT	GGGTCC
3a	Site 7 Nme91-SpRY-NCAC-1	GTTTGTCCCCACAGTCCCCAGGGA	AAAGCC
3a	Site 8 TS71	GCGCAAAGCTGCATCCACCCCCCG	AGGACC
3a	Site 9 Nme99	GCTGGCCGCCAGCCCAGTTGTAGC	ACCGCC
3a	Site 10 TS75	GTTCCCCTTCATTGCGGCGGGCTG	CGGGCC
3b	Site 11 Cj63-AAVS1	GATTGTGCTGTCAGGAGCTCGG	GGGAGTAC
3b	Site 12 Cj56-AAVS1	GAGTAGAGGCGGCCACGACCTG	GTGAACAC
3b	Site 13 Cj672-RNF2	TCCTAATCCCTTGCACAGGGTC	TGGCATAC
3b	Site 14 CJ725-HEK4	GGTCCAAAGCAGGATGACAGGC	AGGGGGCAC
3b	Site 15 Cj57-AAVS1	GGAGTGTGACAGCCTGGGGCCC	AGGCACAC
3b	Site 16 Cj61-AAVS1	AGCTGCAGCACCAGGATCAGTG	AAACGCAC
3b	Site 17 Cj62-AAVS1	GAGGTGGCTAAAGCCAGGGAGA	CGGGGTAC
3b	Site 18 Cj58	GTGGGGTGAGGAGAAGGCTGGG	AGGGATAC
3b	Site 19 Cj59	GCAGGAGGTTTTCCTTGTGGCA	GGAGATAC
3c	Site 20 Sauri-HEK2	GGAACACAAAGCATAGACTGC	GGGG
3c	Site 21 Sauri-HEK3	GGGGCCCAGACTGAGCACGTG	ATGG
3c	Site 22 Sauri-RNF2	GCAGTCATCTTAGTCATTACCT	GAGG
3c	Site 23 Sauri-EMX1	GCTGAGTCCGAGCAGAAGAAGA	AGGG
3c	Site 24 Sauri-FANCF	GATGGAATCCCTTCTGCAGCAC	CTGG
3c	Site 25 HEK4	GTGGCACTGCGGCTGGAGGTG	GGGG
3c	Site 26 GAPDH	CCAGCCCCAGCAAGAGCACAA	GAGG
3c	Site 27 SEC61B	CCTCATCTCCAATATGGTATG	GCGG
3c	Site 28 ABE8e Site 1	GGACAAACCAGAAGCCGCTCC	TGGG
3c	Site 29 ABE8e Site 7	CAGTCATCTTAGTCATTACCT	GAGG
Fig 5b	PCSK9 exon 1 donor SaKKH	GCCTACACCCGCACCTTGGCGC	AGCGGT
Fig 5b	Pcsk9 exon 1 donor SaKKH	GCCATACCTTGGAGCAACGGCG	GAAGGT
Fig 5b	Pcsk9 exon 1 donor Sauri	GCACCCATACCTTGGAGCAACG	GCGG
Fig 5b	Angptl3 exon 6 donor SaKKH	GAGATACCTGAGTAACTTTCTG	GACAGT
Fig 5c	Pcsk9 exon 1 donor Sp	GCCCATACCTTGGAGCAACGG	CGG
SI Fig 1	Sa-W8R	GCCACCGCAGCCACGCAGAGCA	GTGGGT
SI Fig 1	SaKKH-T89A	ATGGAGGAGACCCAGAGGCT	ACAGAT
SI Fig 1	SaKKH-Q259R	GCTCAACTGTCAAGGGAAGGG	CACAGT
SI Fig 1	SaKKH-Q343N	GCCACGAATGCCCAGGACCA	GCCAGT
SI Fig 4a	PCSK9_exon1_start_SaKKH	GGTGCCCATGAGGGCCAGGGG	AGAGGT

SI Fig 4a	PCSK9_exon1_don_Sa_SaKKH	GCACCCGCACCTTGGCGCAGCG	GTGGAA
SI Fig 4a	PCSK9_exon1_don_SaKKH	GCCTACACCCGCACCTTGGCGC	AGCGGT
SI Fig 4a	PCSK9_exon3_acc_SaKKH	GTTGCATGGGGCCAGGATCCGT	GGAGGT
SI Fig 4a	PCSK9_exon4_don_SaKKH	GAGATGGGGGTCTTACCGGGGG	GCTGGT
SI Fig 4a	PCSK9_exon5_acc_SaKKH	GCCTTGGAAAGACGGAGGCAG	CCTGGT
SI Fig 4a	PCSK9_exon5_don_Sa	GCGTGCTTACCTGTCTGTGGAA	GCGGGT
SI Fig 4a	PCSK9_exon5_don_Sa_SaKKH	GACGGCCGTGCTTACCTGTCTG	TGGAAG
SI Fig 4a	PCSK9_exon6_acc_SaKKH	GTCGAGCAGGCCAGCAAGTGT	GACAGT
SI Fig 4a	PCSK9_exon9_acc_SaKKH	GCCCTGCACCAGGCATTGCAGC	CATGAT
SI Fig 4a	PCSK9_exon9_don_Sa	GCCATCCTGCTTACCTGCCCC	ATGGGT
SI Fig 4b	Pcsk9_exon1_start_SaKKH	GTGGGTGCCCATCGGGGCGAGG	AGAGGT
SI Fig 4b	Pcsk9_exon1_don_SaKKH	GCCATACCTTGGAGCAACGGCG	GAAGGT
SI Fig 4b	Pcsk9_exon2_don_SaKKH	GAAGATGGCTCACCAGGCCCA	ACAGGT
SI Fig 4b	Pcsk9_exon3_don_SaKKH	GTACTGGGGACCTTACCAGGGG	AGCGGT
SI Fig 4b	Pcsk9_exon4_acc_SaKKH	GCCATGGGAAGATGGAAGCAG	CCAGGT
SI Fig 4b	Pcsk9_exon8_acc_Sa	GTGTACCTCCAGGCATTGTGGC	TCGGAT
SI Fig 4b	Pcsk9_exon8_don_Sa	GCCATCCTGCTCACCTGTCTC	ATGGGT
SI Fig 4b	Pcsk9_exon9_don_SaKKH	GAGACAGGGTCGGGCACCTCAA	TCCAAT
SI Fig 4c	Angptl3_exon1_start_SaKKH	GTGAGACAAAAAATGCACACAA	TTAAAT
SI Fig 4c	Angptl3_exon1_don_Sa	GTGCTGTGATTTCTACTTACT	TTGAGT
SI Fig 4c	Angptl3_exon1_don_SaKKH	GCTGTGATTTCTACTTACTTTG	AGTGAT
SI Fig 4c	Angptl3_exon3_acc_SaKKH	GTTCTTTTATCAGCTCAGAAAG	ACTGGT
SI Fig 4c	Angptl3_exon4_don_Sa	GTGTTTCCATGGGTTTACCTGA	TTGGGT
SI Fig 4c	Angptl3_exon4_don_SaKKH	GTGGGTTTACCTGATTGGGTAT	CACAGT
SI Fig 4c	Angptl3_exon5_acc_SaKKH	GCTCTCCAGGCAGTCCATGGAC	ATTAAT
SI Fig 4c	Angptl3_exon6_don_SaKKH	GAGATACCTGAGTAACTTTCTG	GACAGT
Extended Data Fig 6	Sauri_Pcsk9_exon1_don_Klienstiver	GCACCCATACCTTGGAGCAACG	GCGG
Extended Data Fig 6	Sauri_Pcsk9_exon1_don_Ran	GCACCCATACCTTGGAGCAACG	GCGG

Supplementary Table 2 | sgRNA scaffolds used in this manuscript.

Fig.	Scaffold sequence
SaABE8e	GTTTTAGTACTCTGTAATGAAAATTACAGAATCTACTAAAACAAGGCAAAATGCCGTGTTTATCTCGTCAACTTGTTGGCGAGATTTTTTT
SaKKH-ABE8e	GTTTTAGTACTCTGTAATGAAAATTACAGAATCTACTAAAACAAGGCAAAATGCCGTGTTTATCTCGTCAACTTGTTGGCGAGATTTTTTT
Nme2 ABE8e	GTTGTAGCTCCCTTTCTCATTTCGGAAACGAAATGAGAACCGTTGCTACAATAAGGCCGTCTGAAAAGATGTGCCGCAACGCTCTGCCCCTTAAAGCTTCTGCTTTAAGGGGCATCGTTTATTTTTTT
CjABE8e	GTTTTAGTCCCTGAAAAGGGACTAAAATAAAGAGTTTGCGGGACTCTGCGGGGTTACAATCCCCTAAAACCGCTTTTTTT
SauriABE8e *in vitro*[2]	GTTTTAGTACTCTGGAAACAGAATCTACTAAAACAAGGCAAAATGCCGTGTTTATCTCGTCAACTTGTTGGCGAGATTTTTTT
SauriABE8e *in vivo*[1]	GTTTTAGTACTCTGTAATGAAAATTACAGAATCTACTAAAACAAGGCAAAATGCCGTGTTTATCTCGTCAACTTGTTGGCGAGATTTTTTT

Supplementary Table 3 l Primers used to amplify genomic DNA for high throughput sequencing.

Binding sequences of primers for genomic amplification are shown, overhangs included for HTS are:
Forward, 5’ extension: ACACTCTTTCCCTACACGACGCTCTTCCGATCTNNNN
Reverse, 5’ extension: TGGAGTTCAGACGTGTGCTCTTCCGATCT

Fig.	Site ID	HTS bind For	HTS bind Rev
1	Pcsk9-W8R	GGCGTCCATGTCCTTCCCGA	ACATGTGCGGCCTCATCAGCCA
3a	Site 1 TS90-GAPDH	GCTCAGAAAAAGGGCCCTGA	GAGATTCAGTGTGGTGGGGG
3a	Site 2 TS89-SEC61B	AACTTTCTATCCGTCCGCGT	GGCTGTAGAGGGAGACAAGC
3a	Site 3 TS88-FANCF	GGGCCTGGAAGTTCGCTAAT	TGGATCGCTTTTCCGAGCTT
3a	Site 4 TS72-LINC01588	ATTGCTCTTTTCTCCGCCCA	TTCACAAAACAGGGGTGGCT
3a	Site 5 TS81-LSP1	GACGTCTTCTCCTGTGGTGG	GGGTGTCTGGCTGGAATCTC
3a	Site 6 Nme10-EMX1	CCCCCGCACTCCTTCTTC	AATCTACCTCCGCGGACCT
3a	Site 7 Nme91-SpRY-NCAC-1	GGCTCCCTCTCCCAGTTACCG	CACCACCATCCGCTCTGCCC
3a	Site 8 TS71	TTCAGGCTGTGAACCTTGGT	CCGCTACCAGCCGACTTTTAA
3a	Site 9 Nme99	GGTGGAAGGTCCCTCCAGA	CCTTCAACCTGACCTGGGAC
3a	Site 10 TS75	ATTGCTCTTTTCTCCGCCCA	TTCACAAAACAGGGGTGGCT
3b	Site 11 Cj63-AAVS1	AAAGAGGAAGCTGTCTCCGC	AGTCTCAGCCAGCCACTTTC
3b	Site 12 Cj56-AAVS1	GCTCTGGGCGGAGGAATATG	TCCGTGCGTCAGTTTTACCT
3b	Site 13 Cj672-RNF2	GATCTGGACGAATAGTGTAGGTTAAG	GCAAGTTGGTTGCTGTCTTTTGG
3b	Site 14 CJ725-HEK4	GAACCCAGGTAGCCAGAGAC	TCCTTTCAACCCGAACGGAG
3b	Site 15 Cj57-AAVS1	GCTATGCAGGGTGGAGGAAG	TGGACTTCGGCTTTTGTCCC
3b	Site 16 Cj61-AAVS1	GGGACTAGAAAGGTGAAGAGCC	GAGGATCCTGGGAGGGAGAG
3b	Site 17 Cj62-AAVS1	GGGACTAGAAAGGTGAAGAGCC	GAGGATCCTGGGAGGGAGAG
3b	Site 18 Cj58	GGTCTTCGTTCCTGGCTGAT	CTTTTCCCTTGGACCCCAGG
3b	Site 19 Cj59	ATCAAGGTCAACACAGGGGG	TCACCATGTTGACCAGGCTG
3c	Site 20 Sauri-HEK2	CCAGCCCCATCTGTCAAACT	CCGATCTTGAATGGATTCCTTGGAAACAATGA
3c	Site 21 Sauri-HEK3	ATGTGGGCTGCCTAGAAAGG	CCCAGCCAAACTTGTCAACC
3c	Site 22 Sauri-RNF2	ACGTCTCATATGCCCCTTGG	ACGTAGGAATTTTGGTGGGA CA
3c	Site 23 Sauri-EMX1	CAGCTCAGCCTGAGTGTTGA	CCGATCTCTCGTGGGTTTGTGGTTGC
3c	Site 24 Sauri-FANCF	CATTGCAGAGAGGCGTATCA	GGGGTCCCAGGTGCTGAC
3c	Site 25 HEK4	GAACCCAGGTAGCCAGAGAC	TCCTTTCAACCCGAACGGAG
3c	Site 26 GAPDH	GCTCAGAAAAAGGGCCCTGA	GAGATTCAGTGTGGTGGGGG
3c	Site 27 SEC61B	AACTTTCTATCCGTCCGCGT	GGCTGTAGAGGGAGACAAGC
3c	Site 28 ABE8e Site 1	AGCCCTCTTTTTATTGGAACTGTG	CCGACTGGTCCACTTACCTA
3c	Site 29 ABE8e Site 7	AACGGAACTCAACCATTAAGCA	CCAACATACAGAAGTCAGGAATGC
4b	PCSK9 exon 1 donor	GCGCACGGCCTCTAGGTCT	GAGGAAACAGCACCGCACCG
4b	Pcsk9 exon 1 donor	GCGTCCATGTCCTTCCCGAG	CCAACCCCAAAGCAACGCCG
4b	Angptl3 exon 6 donor	TCACCTGGGCAGTCACGAAA	ACACCATCAACATAGTGAGGAGAA
SI Fig 1	Pcsk9-W8R	GGCGTCCATGTCCTTCCCGA	ACATGTGCGGCCTCATCAGCCA
SI Fig 1	Pcsk9-T89A	GATGAGGTCATGCTGGGGTCCT	GTAGAACCTTGATGACATAGCCCCG
SI Fig 1	Pcsk9-Q259R	AGGAACTGTTCTCCTCACTCCCA	GCCTGACAGTGAGACCTTGCTTC
SI Fig 1	Pcsk9-Q343N	TGTGGGAACTGGAGTTGGCTAGT	CTGGACGCTCCGATGATGTCCTT
SI Fig 4a	PCSK9_exon1	GAGGAAACAGCACCGCACCG	GAGGAAACAGCACCGCACCG
SI Fig 4a	PCSK9_exon3_acc	ACACCTAGGGTTTGCTGGGTTTCTT	TATGCAGTGGCCCAGCCCTATCA
SI Fig 4a	PCSK9_exon4_don	AGGCTGTGGCTGTGTTTGCT	GGAAACCACCAGCAGGGAGG
SI Fig 4a	PCSK9_exon5	CCAGTGCCTGGGATGTGCTCTG	CAGGAGTACAGCTGCAACGCTC
SI Fig 4a	PCSK9_exon6_acc	TCCAGCCACCTGCTGATTTGT	CCTCCCAGGCCAGGTCCAG
SI Fig 4a	PCSK9_exon9	GCCGGGCCATCACCATCTTT	CACACAGACCTCCCAAGCCC
SI Fig 4b	Pcsk9_exon1	GCGTCCATGTCCTTCCCGAG	CCAACCCCAAAGCAACGCCG

SI Fig 4b	Pcsk9_exon2_don	GATTGAACAAACTGCCCACCGC	CAAGAGCCACAGGCGCTTACTG
SI Fig 4b	Pcsk9_exon3_don	TTGGGAAGAGGACGGGCAGA	TGGGGAGGTGGACAGTCAGG
SI Fig 4b	Pcsk9_exon4_acc	CCGTCTAATGCGTGGGGTGG	GGCACGCTGTTGAAGTCGGT
SI Fig 4b	Pcsk9_exon8	CCCTATATTTTGGGAAGCAGGGC	AGAAAACCTTCCTGTGAGGCCA
SI Fig 4b	Pcsk9_exon9_don	GGGTGCTCCCTCCACCCTAA	AGGTGCTGCTCTCCAGCCAA
SI Fig 4c	Angptl3_exon1	GGAGGGAGAAGTTCCAAATTGCTTA	CAGCTGCAGGAGGCCATTCG
SI Fig 4c	Angptl3_exon3_acc	TGACACACTTACCCAGCATTCCCT	ATTTTGTAGAGAAAACTGCCCGGAT
SI Fig 4c	Angptl3_exon4_don	GCCGACTGCTCTGCCGTTT	CCCAGATTTGCGTGAATAACCATTT
SI Fig 4c	Angptl3_exon5_acc	GCTAAAACCAAACTCAAAACGGGT	CCCATGTCAGTGCAACCAGCTA
SI Fig 4c	Angptl3_exon6_don	TCACCTGGGCAGTCACGAAA	ACACCATCAACATAGTGAGGAGAA
Extended Data Fig 2	Pcsk9_exon1	GCGTCCATGTCCTTCCCGAG	CCAACCCCAAAGCAACGCCG
Extended Data Fig 3a	OT1	AACCCCACCACCTTGGC	ACAGTCCACAGTCTAAGTGCAAA
Extended Data Fig 3a	OT2	TTTGGAGTGGCATATTTGACAGCA	TGTGGCACAGTGGGCCTTTAC
Extended Data Fig 3a	OT3	TCATGCTCTGCCCTGTAGGTA	ATGGCATTCATTTAAGCCTAACTTC
Extended Data Fig 3b	OT1	AAGTATGTTGGGACCCTTGGCTGG	TGGCCTGTTCTACTGACTATGGGG
Extended Data Fig 3b	OT2	GACAGACACAGGGAAGCCTTGGG	AACCTTCCAGGAGAGAGAAACCTGT
Extended Data Fig 3b	OT4	TTCAAGCAATCACGAGACACTCAG	CCCACCACCCAGCAGCTTTATTG
Extended Data Fig 3b	OT5	TCTCAGGCGACCTGGTTTCTGC	TCTGCCAGATGCGTCCGATCA
Extended Data Fig 3b	OT6	GCCAGCCCTGCCTGGAAGTTAG	TGACCTCCGGGATTCTCAGCCC
Extended Data Fig 3b	OT7	GCTTCCTGTCTGCAATTGGGGTCT	AGTAGGTTGCGGGGCTCAGGA
Extended Data Fig 3b	OT9	AACCTCCACGGGGGTATCTGAGG	ACCTGGCAAGTGGGGTACTGG
Extended Data Fig 3b	OT10	GTCTAAATGGGCAAGCAATCCCCT	CCAGGATCCCACAGGGTCCTTCT
Extended Data Fig 3b	OT11	TCCCCAGAGCCCAGGGAATATCA	TGTTGCTCCGATGGAAGGATGGG
Extended Data Fig 3b	OT13	CCACCAGAAGCGCCCCAGAA	GGGGAATCGCCTCCACTGCC
Extended Data Fig 3b	OT14	GTTCTTATTGGCCAGGGAGCCTT	CTCCCCAAGTGACAGGAACCACG
Extended Data Fig 4	cDNA_Canx/Ip90	GGGTCTGGAATATACTCAGGTTCGT	TGACCAGTCTGTTGTGAACAGTG
Extended Data Fig 4	cDNA_Ctnnb1	TGACCTGATGGAGTTGGACATGG	ACTTGCTCTTGCGTGAAGGACT
Extended Data Fig 4	cDNA_Usp38	ACAGGTCTTCCACAGAAGGGC	ACTTGTTTACAAGAGGTGACCAGT
Extended Data Fig 4	cDNA_Aars	AGCCAGAATCCTCCCTGGCAA	CAGAACACCATCCGACTCCCTGT

Supplementary Table 4 l Primer and probe sequences used for ddPCR

**Primer Name**	**Sequence 5' to 3'**
JRD SaCas9 C Term ddPCR For	GCAACTACCTGACAAAGTATAGCAAG
JRD SaCas9 C Term ddPCR Rev	GCTTCAGGCTCAGCTTCACCAC
Gapdh ddPCR Fwd	CGCCCTGATCTGAGGTTAAAT
Gapdh ddPCR Rev	CGGAGCAACAGATGTGTGTA

**Probe Name**	**Sequence 5' to 3'**	**Fluorophore/Quencher**
SaCas9 C Term Probe	CCCACCTGGACATCACCGACGATTACCCTAAC	6-FAM/IB FQ ZEN
Gapdh Probe	AGCCGTGTGACCTTTCTGGATCTG	HEX/IB FQ ZEN

Supplementary Table 5 l Summary of the base editing activity windows of size-minimized ABEs developed in this manuscript and the percentages of targetable genomic adenines**.**

a Window widths used for the calculation of targetable genomic adenines. Window widths are shown with respect to the standard protospacer lengths of each editor, with position 1 being defined as the 5' end of the protospacer.

**Variant**	**Protospacer length (nt)**	**PAM**	**Window (25% max)**
SauriABE	21	NNGG	5-15
SaKKHABE	21	NNNRRT	2-13
CjABE	22	NNNV RYAC	3-7, 9-13, 15
Nme2ABE	24	NNNNCC	6, 9-13, 16-17

b The percentage of genomic adenines targetable with one or more size-minimized ABEs developed in this study using the activity window definitions in (a).

	total_A	targetable_A	total_T	targetable_T	percent targetable
chr1	32546775	26685188	32599119	26731923	81.99612857
chr10	19549602	16072176	19566475	16079439	82.19539756
chr11	18530910	15213431	18548654	15231448	82.10689586
chr12	18869776	15364041	18905105	15390534	81.415412
chr13	15663586	12722581	15718153	12773277	81.24424845
chr14	13041618	10670545	13118537	10736532	81.83084924
chr15	11901023	9791465	11881736	9779219	82.2893761
chr16	10761831	8880058	10847090	8959068	82.55445054
chr17	10711334	8892175	10719812	8897966	83.01068454
chr18	11963020	9779925	12016805	9824978	81.75582182
chr19	5808203	4848031	5816556	4854410	83.46358836
chr2	36905447	30200054	36965508	30246198	81.82681813
chr20	8190084	6756662	8272621	6829290	82.52563598
chr21	5754013	4719119	5767220	4730454	82.01876483
chr22	4583339	3855516	4582999	3855167	84.11955789
chr3	29608996	24099572	29636045	24126868	81.40164845
chr4	29167021	23603218	29177885	23608189	80.91778741
chr5	27049804	22032634	27155675	22123573	81.46078185
chr6	26137405	21311082	26196421	21367050	81.54980299
chr7	23255299	19020621	23243204	19007154	81.78279417
chr8	21395653	17440637	21361906	17416402	81.52251863
chr9	17364609	14209902	17335711	14192377	81.85019331
chrX	17917313	14524778	17926781	14532656	81.06616951
chrY	2994088	2426016	3002884	2431968	81.00728167
all	419670749	343119427	420362902	343726140	81.76405388

Supplementary code I Custom python script for calculating the targetable adenines in the human genome with small ABE8e targetable PAMs.

```
import re
from Bio import SeqIO
import Bio
from Bio.Seq import Seq
import pandas as pd

def is_targetable(sequence, A_position, window, PAM_seq, protospacer_length):
        #convert PAM to regex PAM
        regex_PAM =
PAM_seq.replace('N','[ATGC]').replace('R','[AG]').replace('Y','[CT]').replace('V
','[AGC]')
        is_targetable = 0
        for coords in window:
                test_for_PAM = sequence[A_position + (protospacer_length -
coords[1]) + 1:A_position + (protospacer_length - coords[0]) + 1 + len(PAM_seq)]
                if [m.start() for m in re.finditer(regex_PAM, test_for_PAM)]:
                        is_targetable = 1
        return is_targetable

genome_fa = '/Volumes/Storage/AR/genome_builds/hg38/hg38.fa'

records = SeqIO.to_dict(SeqIO.parse(genome_fa,'fasta'))

keys = records.keys()

#store genome data as dict = {'chrN': (seq, reverse complement seq)}
sequences = {key: (str(records[key].seq),
str(records[key].seq.reverse_complement())) for key in records.keys()}

#output targetable counts as dict = {'chrN': {'targetable_A': int}, {'total_A':
int}, {'targetable_T': int}, {'total_T'}: int}
output = {key: {} for key in records.keys()}

#free up some memory
del records

#iterate over chromosomes and populate output dict
for chromosome in keys:
        print('Tabulating ' + str(chromosome) + '...')
        #do forward (sense) seq first; findall As
        A_positions = [m.start() for m in re.finditer('A',
sequences[chromosome][0])]
        #update total_A value
        output[chromosome]['total_A'] = len(A_positions)
        #calculate targetable As
        targetable_As = []
        for A_position in A_positions:
                targetable_As.append(0)
                if is_targetable(sequences[chromosome][0], A_position, [[1,12]],
'NNNRRT', 20):
                        targetable_As[-1] = 1
                elif is_targetable(sequences[chromosome][0], A_position,
[[4,14]], 'NNGG', 20):
                        targetable_As[-1] = 1
                elif is_targetable(sequences[chromosome][0], A_position, [[2,2],
[5,9], [12,13]], 'NNNNCC', 20):
                        targetable_As[-1] = 1
                elif is_targetable(sequences[chromosome][0], A_position, [[1,5],
[7,11], [13,13]], 'NNNVRYAC', 20):
                        targetable_As[-1] = 1
        #update targetable_A value
```

```
        output[chromosome]['targetable_A'] = sum(targetable_As)
        #do reverse complement seq next
        T_positions = [m.start() for m in re.finditer('A',
sequences[chromosome][1])]
        output[chromosome]['total_T'] = len(T_positions)
        targetable_Ts = []
        for T_position in T_positions:
                targetable_Ts.append(0)
                if is_targetable(sequences[chromosome][1], T_position, [[1,12]],
'NNNRRT', 20):
                        targetable_Ts[-1] = 1
                elif is_targetable(sequences[chromosome][1], T_position,
[[4,14]], 'NNGG', 20):
                        targetable_Ts[-1] = 1
                elif is_targetable(sequences[chromosome][1], T_position, [[2,2],
[5,9], [12,13]], 'NNNNCC', 20):
                        targetable_Ts[-1] = 1
                elif is_targetable(sequences[chromosome][1], T_position, [[1,5],
[7,11], [13,13]], 'NNNVRYAC', 20):
                        targetable_Ts[-1] = 1
        output[chromosome]['targetable_T'] = sum(targetable_Ts)

#calculate combined totals for all chromosomes
all_total_A = 0
all_total_T = 0
all_targetable_A = 0
all_targetable_T = 0
for chromosome in output.keys():
        all_total_A += output[chromosome]['total_A']
        all_targetable_A += output[chromosome]['targetable_A']
        all_total_T += output[chromosome]['total_T']
        all_targetable_T += output[chromosome]['targetable_T']

output['all'] = {'total_A': all_total_A, 'targetable_A': all_targetable_A,
'total_T': all_total_T, 'targetable_T': all_targetable_T}

output_df = pd.DataFrame.from_dict(output, orient='index')
output_df['percent targetable'] = (output_df['targetable_A'] +
output_df['targetable_T'])/(output_df['total_A'] + output_df['total_T'])*100

output_df.to_csv('2022-06-03/genome-wide_BE_search_Sauri-SaKKH-Nme2-Cj.csv')
```

Supplementary references

1. Kleinstiver, B. P., et al. (2015). "Broadening the targeting range of Staphylococcus aureus CRISPR-Cas9 by modifying PAM recognition." Nature Biotechnology **33**(12): 1293-1298.
2. Ran, F. A., et al. (2015). "In vivo genome editing using Staphylococcus aureus Cas9." Nature **520**(7546): 186-191.
