# Supplementary material for: Efficient in vivo base editing via single adeno-associated viruses with size-optimized genomes encoding compact adenine base editors
Source: Nat Biomed Eng. 2022 Jul 28;6(11):1272–83. doi: 10.1038/s41551-022-00911-4 (PMC9652153; doi:10.1038/s41551-022-00911-4)
Supplement: Supplementary file 2 — Reporting Summary [file 41551_2022_911_MOESM2_ESM.pdf]

nature portfolio

Corresponding author(s): David R. Liu

Last updated by author(s): Jun 14, 2022

# Reporting Summary

Nature Portfolio wishes to improve the reproducibility of the work that we publish. This form provides structure for consistency and transparency in reporting. For further information on Nature Portfolio policies, see our Editorial Policies and the Editorial Policy Checklist.

## Statistics

For all statistical analyses, confirm that the following items are present in the figure legend, table legend, main text, or Methods section.

| n/a | Confirmed | |
|---|---|---|
| ☐ | ☒ | The exact sample size (*n*) for each experimental group/condition, given as a discrete number and unit of measurement |
| ☐ | ☒ | A statement on whether measurements were taken from distinct samples or whether the same sample was measured repeatedly |
| ☐ | ☒ | The statistical test(s) used AND whether they are one- or two-sided<br>*Only common tests should be described solely by name; describe more complex techniques in the Methods section.* |
| ☒ | ☐ | A description of all covariates tested |
| ☐ | ☒ | A description of any assumptions or corrections, such as tests of normality and adjustment for multiple comparisons |
| ☐ | ☒ | A full description of the statistical parameters including central tendency (e.g. means) or other basic estimates (e.g. regression coefficient) AND variation (e.g. standard deviation) or associated estimates of uncertainty (e.g. confidence intervals) |
| ☐ | ☒ | For null hypothesis testing, the test statistic (e.g. *F*, *t*, *r*) with confidence intervals, effect sizes, degrees of freedom and *P* value noted<br>*Give P values as exact values whenever suitable.* |
| ☒ | ☐ | For Bayesian analysis, information on the choice of priors and Markov chain Monte Carlo settings |
| ☒ | ☐ | For hierarchical and complex designs, identification of the appropriate level for tests and full reporting of outcomes |
| ☒ | ☐ | Estimates of effect sizes (e.g. Cohen's *d*, Pearson's *r*), indicating how they were calculated |

*Our web collection on statistics for biologists contains articles on many of the points above.*

## Software and code

Policy information about availability of computer code

| | |
|---|---|
| Data collection | HTS data were collected with Illumina MiSeq. Histology slides were digitized with a Leica Aperio Slide Scanner. |
| Data analysis | HTS data were analysed with CRISPResso2. The targetable genome was calculated via a custom script (provided in the Supplementary Information). |

For manuscripts utilizing custom algorithms or software that are central to the research but not yet described in published literature, software must be made available to editors and reviewers. We strongly encourage code deposition in a community repository (e.g. GitHub). See the Nature Portfolio guidelines for submitting code & software for further information.

## Data

Policy information about availability of data

All manuscripts must include a data availability statement. This statement should provide the following information, where applicable:

- Accession codes, unique identifiers, or web links for publicly available datasets
- A description of any restrictions on data availability
- For clinical datasets or third party data, please ensure that the statement adheres to our policy

The data supporting the results in this study are available within the paper and its Supplementary Information. All unmodified reads for sequencing-based data are available from the NCBI Sequence Read Archive, under accession number PRJNA798016. AAV genome sequences are provided in the Supplementary Information.

Key plasmids from this work will be available from Addgene (depositor: David R. Liu), and other plasmids and raw data are available from the corresponding author on request.

## Human research participants

Policy information about studies involving human research participants and Sex and Gender in Research.

| | |
|---|---|
| Reporting on sex and gender | The study did not involve human research participants. |
| Population characteristics | — |
| Recruitment | — |
| Ethics oversight | — |

Note that full information on the approval of the study protocol must also be provided in the manuscript.

# Field-specific reporting

Please select the one below that is the best fit for your research. If you are not sure, read the appropriate sections before making your selection.

☒ Life sciences ☐ Behavioural & social sciences ☐ Ecological, evolutionary & environmental sciences

For a reference copy of the document with all sections, see nature.com/documents/nr-reporting-summary-flat.pdf

# Life sciences study design

All studies must disclose on these points even when the disclosure is negative.

| | |
|---|---|
| Sample size | Sample sizes were chosen in accordance with standards in the field that have historically been sufficient to develop and validate genome-editing technologies. |
| Data exclusions | No data were excluded from analysis. |
| Replication | All replicates reported are independent. All attempts at reproducibility succeeded, as measured by at least two or three positive results. |
| Randomization | Mice were assigned to groups randomly and were age-matched between conditions. No covariates were controlled for. |
| Blinding | All HTS data were analysed by an unblinded operator by using an automated CRISPResso2 script with limited experimenter intervention. Plasma analytes were analysed by an unblinded operator. Histology was analysed by a pathologist blinded to treatment conditions. |

# Reporting for specific materials, systems and methods

We require information from authors about some types of materials, experimental systems and methods used in many studies. Here, indicate whether each material, system or method listed is relevant to your study. If you are not sure if a list item applies to your research, read the appropriate section before selecting a response.

### Materials & experimental systems

| n/a | Involved in the study | |
|---|---|---|
| ☒ | ☐ | Antibodies |
| ☐ | ☒ | Eukaryotic cell lines |
| ☒ | ☐ | Palaeontology and archaeology |
| ☐ | ☒ | Animals and other organisms |
| ☒ | ☐ | Clinical data |
| ☒ | ☐ | Dual use research of concern |

### Methods

| n/a | Involved in the study | |
|---|---|---|
| ☒ | ☐ | ChIP-seq |
| ☒ | ☐ | Flow cytometry |
| ☒ | ☐ | MRI-based neuroimaging |

## Eukaryotic cell lines

Policy information about cell lines and Sex and Gender in Research

| | |
|---|---|
| Cell line source(s) | HEK293T, Neuro-2A and NIH-3T3 cells were obtained from ATCC. |

| | |
|---|---|
| Authentication | The cell lines were not authenticated. |
| Mycoplasma contamination | The cell lines tested negative for mycoplasma (tested periodically during use). |
| Commonly misidentified lines (See ICLAC register) | No commonly misidentified cell lines were used. |

## Animals and other research organisms

Policy information about studies involving animals; ARRIVE guidelines recommended for reporting animal research, and Sex and Gender in Research

| | |
|---|---|
| Laboratory animals | Mice were of either the C57BL/6 or C57BL/6 background lines containing the human PCSK9 gene, and were sourced from The Jackson Laboratory. The mice were 6–12 weeks of age, and weighed roughly 20–25 g at the time of injection. |
| Wild animals | The study did not involve wild animals. |
| Reporting on sex | Both male and female mice were used for each condition. Sex was not disaggregated for analysis. |
| Field-collected samples | The study did not involve samples collected from the field. |
| Ethics oversight | The Broad Institute and the University of Pennsylvania IACUCs provided ethical oversight for all animal experiments. |

Note that full information on the approval of the study protocol must also be provided in the manuscript.
